# Further characterisation of immortalised human lymphatic endothelial cells to explore their transcriptomic profile and VEGFC response

**DOI:** 10.1038/s41598-025-28510-8

**Published:** 2025-12-13

**Authors:** Kazim Ogmen, Ruby Moy, Sara E. Dobbins, Lotte van den Bent, Onno Kranenburg, Jeroen Hagendoorn, Alan Pittman, Pia Ostergaard, Silvia Martin-Almedina

**Affiliations:** 1 School of Health & Medical Sciences, City St George’s, University of London, Cranmer Terrace, London, SW17 0RE UK; 2 Laboratory of Translational Oncology & Dept. of Surgical Oncology, University Medical Center Utrecht, Utrecht, The Netherlands

**Keywords:** Biological techniques, Biotechnology, Cell biology, Molecular biology

## Abstract

In vitro modelling relies on the availability of suitable cell types that accurately represent the organs under study. In lymphatic research, human dermal lymphatic endothelial cells represent the “gold standard”, even though they lose their identity and proliferative capacity over time. A recently established immortalised lymphatic endothelial cell line (imLEC) could become a promising new tool for lymphatic disease modelling. We further characterised this cell line by comparing imLECs and HDLECs in terms of the expression of proteins essential for correct lymphatic function, and the proliferation, migration and sprouting responses to vascular endothelial growth factor C (VEGFC). We show similarities in the expression of lymphatic markers and VEGFC-driven cellular responses, supporting imLECs can retain their VEGFC-driven lymphangiogenic capacity without losing their lymphatic identity. RNA sequencing, however, revealed certain transcriptional differences in genes regulating lymphatic function in health and disease, highlighting the need for further validation at single gene level or specific lymphatic-associated signalling pathways. We acknowledge these limitations should be considered in future applications. Nonetheless, we believe that imLECs represent a useful model for the development of gene editing techniques allowing better modelling of lymphatic disease-associated genetic variants, ensuring long-term culture and providing higher reproducibility in genotype–phenotype validation analyses.

**Supplementary Information:**

The online version contains supplementary material available at 10.1038/s41598-025-28510-8.

## Introduction

Primary Lymphatic Anomalies (PLA) are a group of heterogeneous conditions characterised by tissue fluid retention and swelling in any part of the body due to inborn errors of lymphatic development that compromise lymphatic function^1^. To date, a genetic diagnosis is typically only possible in less than 26% of cases due to this heterogeneity^2,3^. Whole exome and genome sequencing also lead to the discovery of many novel variants of unknown significance (VUS), that must be functionally validated before a disease-causing molecular diagnosis can be given to patients^4–6^.

Investigations into the pathogenesis of newly identified PLA variants rely on robust cell models that allow their reproducible functional assessment. At present, commercially available human dermal lymphatic endothelial cells (HDLECs) are a widely accepted in vitro model in the lymphatic research field. However, these cells tend to lose their lymphatic identity, entering senescence after several passages. Furthermore, they show batch-to-batch variation due to their single-donor origin, and limited efficiency of ectopic gene expression often inducing cell toxicity impacting downstream analysis. CRISPR/Cas gene editing tools are a promising methodology for the interrogation of PLA variant pathogenesis, allowing the modification of the intrinsic DNA and providing a more genetically accurate disease model. However, due to their limited lifespan, HDLECs may be less suitable for long-term clonal selection in CRISPR/Cas gene editing protocols.

Human derived immortalised cell lines have been established from different endothelial beds to overcome these limitations, retaining morphological and functional characteristics over long culture, and providing an exciting array of new cell lines available for studying biologically relevant processes or mechanisms of disease^7–13^.

Telomerase gene expression has been used to generate several immortalised lymphatic endothelial cell lines derived from human dermal microvascular endothelial cells (HDMVECs)^14,15^. More recently, Frenkel and colleagues combined two lentiviral cassettes containing human telomerase reverse transcriptase subunit (hTERT) and GFP-fused BMI-1 (a component of the Polycomb Repressive Complex I which up-regulates hTERT expression) to generate long-lived lymphatic endothelial cells from human microvascular endothelial cells (dLyNeo-Der Lym); referred to as immortalised lymphatic endothelial cells (imLECs) in this study. They demonstrated that these imLECs have increased life span in vitro whilst retaining LEC morphology and expression of lymphatic markers, such as CD31, PROX1, podoplanin and LYVE-1 for over 12 months in culture^16^.

To build on the work of Frenkel and colleagues, the aim of the present study is to further characterise the expression levels of lymphatic markers in imLECs, especially some of those also known to be implicated in PLA, and to assess their responsiveness to Vascular Endothelial Growth Factor C (VEGFC), the main growth factor promoting lymphangiogenesis, in comparison to HDLECs (commercially obtained from PromoCell). If they prove to be a suitable model for in vitro characterisation, the use of long-lived imLECs would allow us to utilise gene editing techniques such as CRISPR/Cas to better model pathogenic variants found in PLA-associated genes, and to provide higher reproducibility for downstream transcriptomic and cell-based assays.

We performed a combination of immunofluorescence assays and flow cytometry to study the expression and localisation of well-known lymphatic endothelial cell markers including PLA-associated proteins in both early and late passages of imLECs and HDLECs. Immunofluorescence and western blots were also used to determine whether long-term culture impacts on imLECs’ senescence. In addition to this, RNA-sequencing was performed to investigate transcriptional signatures in both cell types. Label-free high contrast imaging using Livecyte microscopy was used for the quantification of single-cell proliferation and migration in response to VEGFC. Finally, we tested whether VEGFC stimulation effects 3D spheroid-based sprouting in both cell models in early and late passages.

In summary, we found that the long-term expression of lymphatic endothelial markers, including EPHB4, FOXC2 and ERG, proteins also associated with PLA, can be sustained in imLECs. In addition, our findings suggest 100 ng/mL VEGFC induces proliferative and migratory responses in imLECs, albeit significant responses were also observed even at basal conditions (in the absence of VEGFC). Importantly, we showed that imLECs survive longer in vitro without reaching senescence compared to HDLECs, and with VEGFC responsiveness sustained even at late passages (over five months in culture). Finally, RNA-sequencing shows high levels of correlation but slightly different PLA-associated gene transcription signatures in imLECs and HDLECs. We conclude that the suitability of imLECs for in vitro experimentation investigating single genes or signalling pathways in the context of VEGFC stimulation is promising. However, imLECs should be further investigated and carefully validated for PLA disease modelling and the study of other lymphatic-specific functional pathways.

## Results and discussion

### imLECs retain long-term expression of lymphatic endothelial markers including the PLA-associated proteins EPHB4, FOXC2 and ERG without reaching replicative senescence

We first confirmed that imLECs retain their GFP-tag and lymphatic endothelial cell morphology. By using live-staining flow cytometry, we found that > 92% of imLECs express the GFP-tag (Supplementary Fig. 1A ) due to the presence of the GFP-BMI-1 vector^16^. PECAM1 (CD31) expression was found to be comparable between HDLECs (70.16%) and imLECs (72.93%) by flow cytometry (Supplementary Fig. 1A ), and both cell types grew with a cobblestone appearance, as can be seen by immunofluorescence staining with positive CD31 and Prospero homeobox protein 1 (PROX1) expression, confirming a lymphatic endothelial phenotype (Supplementary Fig. 1B ).

**Fig. 1:**
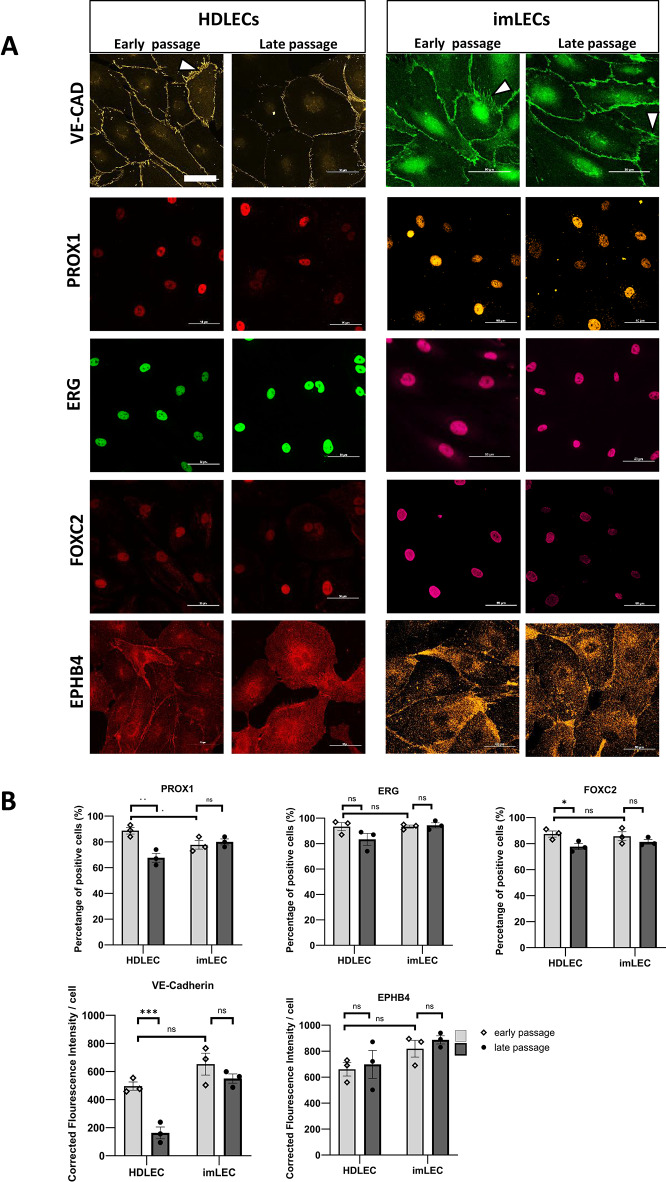
Expression of lymphatic markers and PLA-associated proteins in imLECs. (**A**) Representative images of HDLECs (passage 3 and 7) and imLECs (passage 15 and 39) comparing lymphatic cell marker expression between early and late passage cells analysed by immunofluorescence and confocal microscopy. For labelling PROX1, ERG and FOXC2, secondary antibody Alexa Fluor™ 647 Donkey anti-Goat IgG, anti-Rabbit IgG and anti-Mouse IgG were used, respectively (to prevent signal bleeding from/into DAPI channel). Anti- VE-Cadherin + Alexa Fluor™ 546 Donkey anti-Goat IgG, and anti-EPHB4 + Alexa Fluor™ 555 Donkey anti-Goat IgG were used separately. DAPI counter staining was done for quantification but not shown. All colours in the figure are pseudo-colours. Arrowheads mark active VE-Cadherin in cell membranes. Scale bars = 50 µm. (**B**) Percentage of positive cells for PROX1, ERG and FOXC2 were quantified by manually counting 50 cells per independent repeat and averages taken of each repeat. Total numbers of cells were calculated based on DAPI staining (not shown). For EPHB4 and VE-Cadherin, corrected fluoresce intensity (CFI) per cell was calculated by subtracting background signal from the primary antibody signal using imageJ (JACOB plugin). Images were taken at 60 × magnification and analysis done based on 5 regions of interests (approx. 15–20 cells each) of 3 independent experiments with Nikon A1R confocal microscope. Error bars indicate mean ± S.E., ns means nonsignificant and ***p < 0.001; **p < 0.01 and *p < 0.05 significant based on one-way ANOVA with Tukey’s post hoc test or two-tailed unpaired t test.

Immunofluorescence staining revealed positive expression of VE-Cadherin in the cell membrane of imLECs (Fig. 1A) without significant differences between early and late passages, measured by fluorescence intensity (Fig. 1B). In contrast, in HDLECs at late passage we observed a severe reduction of VE-Cadherin intensity and the loss of the typical serrated junction morphology resembling an active junction configuration, as observed in endothelial cells of different origins^17^. Positive expression of PROX1 was found in the nucleus of imLECs albeit with a significant reduction (12%) of positive cells compared to early HDLECs. Like VE-Cadherin, PROX1 also showed consistent expression between early and late passages in imLECs, whereas both markers were found to be significantly downregulated in late passages of HDLECs compared to early passages, possibly contributing to identity loss as primary cells age (Fig. 1A and 1B).

Next, we determined the expression and localisation of three proteins (EPHB4^18^, FOXC2^19^ and ERG^20^) known to also be associated with PLA in early and late passages of imLECs and HDLECs. We found that the subcellular distribution and staining patterns were similar between both cell types (Fig. 1A). Quantification of EPHB4, FOXC2 and ERG protein expression revealed that there were no noticeable differences between early and late passages of imLECs, and no differences when compared to HDLECs for all proteins analysed. Only the percentage of FOXC2 positive cells was reduced in HDLECs late passage compared to early passage, while ERG and EPHB4 remained unchanged (Fig. 1B). Although the process of immortalization could have impacted the expression of tissue specific cell markers, Frenkel and colleagues extensively confirmed the lymphatic identity of imLECs confirming the expression of CD31, PROX1, podoplanin and LYVE-1. In addition, we have shown here the long-term expression of another three key lymphatic markers, EPHB4, FOXC2 and ERG, and highlighted their comparable subcellular localization and expression levels between imLECs and HDLECs.

Primary cells have a limited life span before they reach replicative senescence, which can be a major disadvantage especially when investigating the long-term impact of genetic variants at functional level. To assess whether imLECs may enter replicative senescence after long-term culture, we first quantified the percentage of multinuclear cells between early and late passages in both cell types. Senescent cells frequently contain multiple nuclei in a single cell body; therefore, appearance of multinucleated cells is associated with senescence^21,22^.

**Fig. 2:**
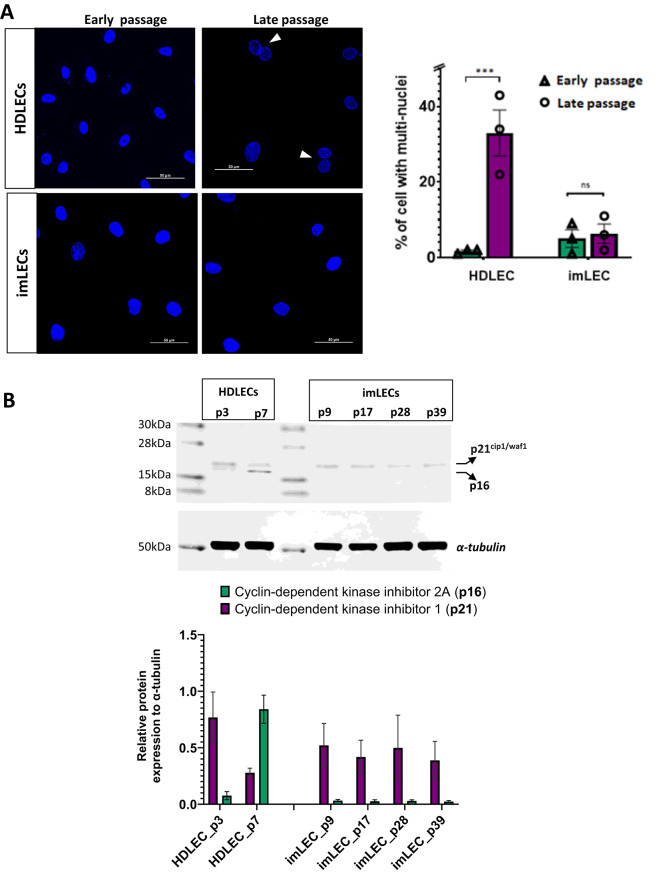
Immortalised lymphatic cells show markers of senescence evasion after long-term culture. (**A**) Left: representative images comparing the presence of multi-nucleated cells (arrowheads) between early and late passages of both imLECs (passage 9 and 39) and HDLECs (passage 3 and 7). Images of fixed cells stained with DAPI were taken at 60 × magnification with a Nikon A1R confocal microscope. Images were acquired from five regions of interest across three independent experiments and analysed using imageJ. Scale bar 50 µm. Right: bar chart represents the mean of *n* = 3 independent experiments, indicating 30% increase in multinucleated cells in late HDLECs passages but no significant change between imLECs. Error bars indicate mean ± S.E., ns means nonsignificant and ***p < 0.001; **p < 0.01 and *p < 0.05 significant based on one-way ANOVA with Tukey’s post hoc test. (**B**) Top panel: representative image of immunoblot indicating expression of p21^cif1/waf1^ and p16 in HDLECs (passage 3 and 7) and in imLECs (passages 9, 17, 28 and 39) with α-tubulin used as internal loading control. Molecular markers (kDa) are labelled on the left of the blot. Bottom panel: bar chart indicates quantification of p21^cif1/waf1^ and p16^INK4a^ relative to α-tubulin. Error bars indicate mean (*n* = 2 independent experiments), ± stdev (standard deviation).

HDLECs yielded 32% more multinuclear cells as they grow in culture, whereas in imLECs no significant difference was found after more than five months in culture (Fig. 2A). To confirm this further, we then measured the levels of two cell cycle regulators, p21^cip1/waf1^ (CDKN1A) and p16 (CDKN2A), where translational expression is closely associated with features of senescence in human endothelial cells^23^. Densitometric analysis of western blots in HDLECs revealed a 70% increase in total p16 expression in late passage compared to early passage, whereas in imLECs, protein expression of p16 was undetectable throughout all passages analysed (Fig. 2B). On the other hand, p21^cip1/waf1^ expression was relatively higher compared to p16 in early passage HDLEC and consistent throughout in all imLEC passages analysed.

Although p21 and p16 are both associated with the induction of senescence, they do not always go together since their expression levels can vary depending on multiple factors such as the stimuli inducing senescence or the cell type under study^24^. In fibroblasts, for instance, p21^cip1/waf1^ decreases after senescence is established while upregulation of p16 may be essential for maintenance of the senescent-cell-cycle arrest^25^. In our context, our results suggest that increased expression of p16 could be used as a marker of lymphatic cell senescence in vitro. Regarding the relatively higher expression of p21 observed in both imLECs and HDLECs, it is known that endothelial tip cells with high VEGFR/ERK signalling strongly upregulate p21, suggesting a mechanistic role for p21 in controlling physiological proliferation^26^. Considering the conserved cellular responses to VEGF growth factors in blood and lymphatic endothelial cells, this may explain the observed expression of p21 in both imLECs and HDLECs. Although the immunoblot results from two independent cell donors support the notion of senescence evasion in imLECs compared to HDLECs, this conclusion should be further strengthened through an increased number of biological replicates to enable robust statistical analysis.

Overall, we have seen that in HDLECs elevated levels of p16 correlates with senescent multinucleated cells, whereas imLECs did not show those cellular senescence markers even after 39 passages (five months) in vitro. Most importantly, expression of selected lymphatic-specific proteins including some also associated with PLA were retained throughout several months in culture.

### RNA-sequencing shows a high level of correlation but slightly different PLA-associated gene transcription signatures in imLEC and HDLECs

Frenkel and colleagues used RNA-sequencing to compare transcriptomic signatures between their generated imLECs and naïve HDMVECs (from Lonza). Since HDLECs obtained from PromoCell are a widely used lymphatic cell model, we aimed to address the transcriptional similarities and differences between our HDLECs from PromoCell and imLECs at early and late passages, by performing RNA-sequencing. FASTQ file alignment provided an average uniquely mapped alignment score of 94.5%, with the lowest being 92.4%, confirming good alignment quality (data not shown). Principal Component Analysis (PCA) was used to generate two PCA plots with samples coloured by either passage or cell-type. We observed three main clusters, with HDLECs clustering together, regardless of passage and imLECs having two clusters differentiated by passage (Supplementary Fig. 2A ).

**Fig. 3:**
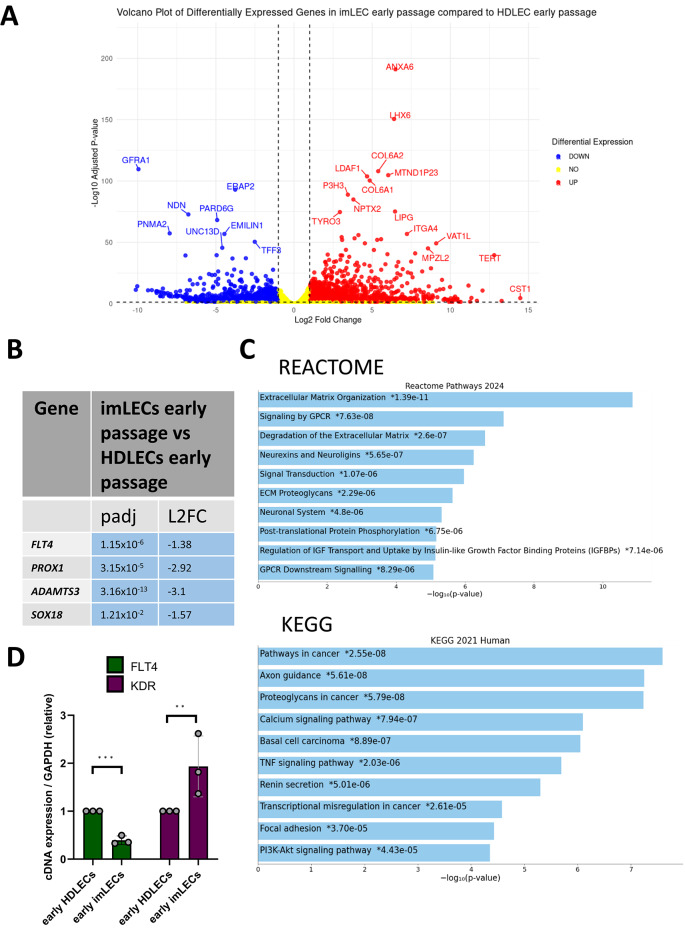
Comparative Bulk RNA-Sequencing between early HDLECs (p3) and early imLECs (p11). (**A**) Volcano plot showing genes that are significantly differentially expressed in early imLECs compared to early HDLECs. Significance is classed as genes that have a padj value < 0.05 and a log fold change value of > 1 or < -1. Padj has been corrected for multiple hypothesis testing using the Benjamini–Hochberg method to control the false discovery rate. Genes in blue are significantly downregulated, and in red significantly upregulated. Yellow means non-significant. (**B**) Table showing PLA-associated genes that are significantly differentially expressed (log fold change value of > 1 or < -1, padj < 0.05). All diagnostic-grade genes on the PLA gene panel were assessed. Squares are coloured blue if downregulated and orange if upregulated, with padj value and log2 fold-changes (L2FC) included. (**C**) Bar chart of the top hits from the Reactome-Pathways_2024 (top) and KEGG_2021_Human gene set library (bottom). KEGG database was developed by Kanehisa laboratories^36–38^. The top 10 enriched terms for the input gene set are displayed based on the -log10(p-value), with the actual p-value shown next to each term. The term at the top has the most significant overlap with the input query gene set. All significantly differentially expressed genes were input. An asterisk (*) next to a p-value indicates the term also has a significant adjusted p-value (< 0.05). (**D**) RNA-sequencing validation of *FLT4* and *KDR* expression levels by RT-qPCR between early HDLECs and early imLECs. Error bars indicate mean (n = 3 independent experiments), ± SE (standard error). Two-tailed unpaired Student’s t-test.

Spearman’s rank correlation was done to identify any significant difference in overall expression between the whole transcriptome of imLECs and HDLECs. We observed a very high level of correlation between all samples (Spearman correlation coefficient = 0.99) (Supplementary Fig. 2B , left panel). Given our special interest in Primary Lymphatic Anomalies, we were keen to investigate the correlation between samples focusing on genes on the PLA gene panel list (see Methods). We also extended our analysis to additional lymphatic markers (e.g. *PROX1*) not yet associated with lymphatic disease, but key regulators of lymphatic development and function. This analysis revealed again a high correlation between all samples analyzed (Supplementary Fig. 2B , right panel). Next, we used DESeq2 to assess the differentially expressed genes between the samples. For our first analysis, we used early passage HDLECs as the baseline/control sample and early passage imLECs as the ‘treated’ samples, which provides us with genes that are differentially expressed in imLECs compared to HDLECs. We hypothesised that, due to the high correlation observed in the previous analysis, there would be a minimal number of differentially expressed genes between cell types at the early passages. We found that out of 29,353 genes with non-zero total read count, 4.74% (1,391) were significantly upregulated and 3.07% (902) were significantly downregulated, with some of these genes having a marked high log fold change and significant *P*-values (Fig. 3A, Supplementary Table 1).

As imLECs have been transformed with hTERT and BMI-1, which prevent differentiation whilst promoting proliferation, they are generally considered stable with no sign of transformation or drifting phenotype^27^. However, we did find transcriptional changes in, for example, *NDRG1*, an anti-oncogenic gene^28^, which was found to be significantly downregulated in imLECs (Log2FC = -1.808334708, Supplementary Table 1). Another example is *TYRO3*, believed to promote survival, chemoresistance and motility in tumour cells^29^, which we found significantly upregulated (Log2FC = 2.933588494, Supplementary Table 1). However, we also detected a few differences in the expression of genes important for lymphatic development and function, including some also associated with PLA. While the majority of the lymphatic-related genes (41 out of 45 analyzed) showed no difference in expression, *FLT4* (encoding VEGFR3), *PROX1, ADAMTS3* and *SOX18* were downregulated in early passage imLECs compared to HDLECs (Fig. 3B). The downregulation of *FLT4* was validated through RT-qPCR (Fig. 3D**).**

**Fig. 4:**
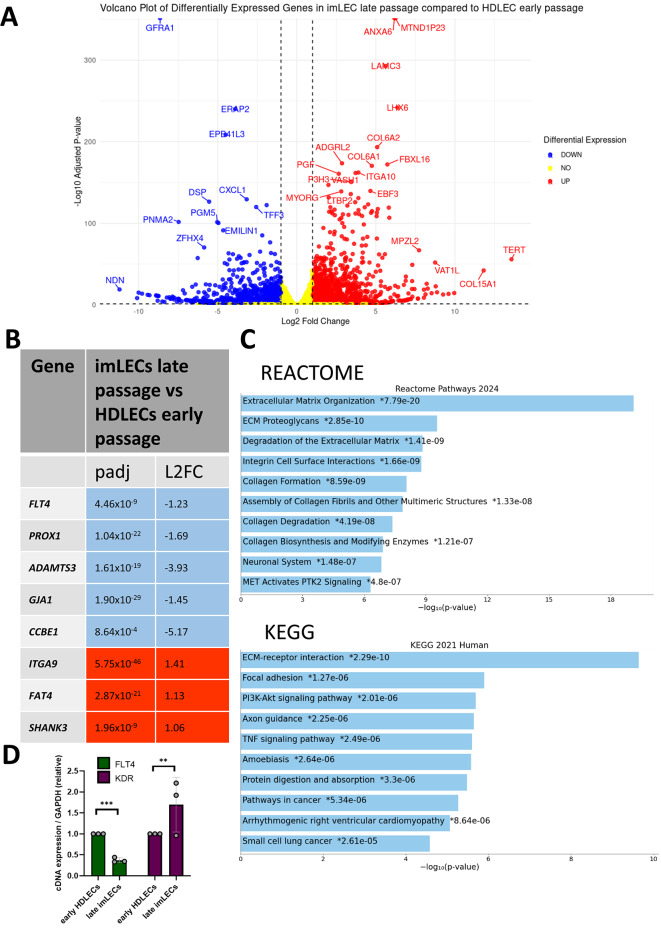
Comparative Bulk RNA-Sequencing between early HDLECs (p3) and late imLECs (p22). (**A**) Volcano plot showing genes that are significantly differentially expressed in late imLECs compared to early HDLECs**.** Significance is classed as genes that have a padj value < 0.05 and a log fold change value of > 1 or < -1. Padj has been corrected for multiple hypothesis testing using the Benjamini–Hochberg method to control the false discovery rate. Genes in blue are significantly downregulated, and in orange if significantly upregulated. Yellow means non-significant. (**B**) Table showing PLA-associated genes that are significantly differentially expressed (log fold change value of > 1 or < -1, padj < 0.05). All diagnostic-grade genes on the PLA gene panel were assessed. Squares are coloured blue if downregulated and red if upregulated, with padj value and log2 fold-changes (L2FC) included. (**C**) Bar chart of top hits from the Reactome-Pathways_2024 (top) and KEGG_2021_Human gene set library (bottom). KEGG database was developed by Kanehisa laboratories^30–32^. The top 10 enriched terms for the input gene set are displayed based on the -log10(p-value), with the actual p-value shown next to each term. The term at the top has the most significant overlap with the input query gene set. All significantly differentially expressed genes were input. An asterisk (*) next to a p-value indicates the term also has a significant adjusted p-value (< 0.05). (**D**) RNA-sequencing validation of *FLT4* and *KDR* expression levels by RT-qPCR between early HDLECs and late imLECs. Error bars indicate mean (n = 3 independent experiments) ± SE (standard error). Two-tailed unpaired Student’s t-test.

Next, we repeated the analysis but comparing early passage HDLECs and late passage imLEC. This analysis revealed a similar picture, with out of 28,897 genes with a non-zero total read count, 5.31% (1,536) were significantly upregulated and 4.22% (1,220) significantly downregulated (Fig. 4A, Supplementary Table 2). While again most lymphatic associated genes (37 out of 45 analyzed) showed no difference in expression, we found a significant downregulation of *FLT4*, *PROX1, ADAMTS3, GJA1* and *CCBE1* and upregulation of *ITGA9, FAT4* and *SHANK3* (Fig. 4B). Again, we validated the downregulation of *FLT4* through RT-qPCR (Fig. 4D**).**

Finally, we questioned if there are any genes expressed in HDLECs that are not expressed in imLECs. To do this, we used DESeq2 to normalise the raw count data (Supplementary Table 3). Filtering for genes not expressed in imLECs (selecting normalised imLEC genes with count = 0 and HDLEC genes with counts > 100), 5 genes (*HLA-DPA1, SCN3A, BHMT2, TC2N* and *POSTN)* were identified. The function of these genes ranges from methyl metabolism to ECM remodelling (EnrichR webserver^33^).

We conclude that at least at the transcriptional level there is a small number of differentially expressed genes in early and late passage imLECs compared to early passage HDLECs, some of which, e.g. *PROX1*, *VEGFR3*, *SOX18* and *ADAMTS3,* are involved in the differentiation and maintenance of the lymphatics^34^. Changes in their expression levels are expected to influence major VEGFR3 downstream signaling cascades such as the AKT and the ERK signaling pathways. This could impact lymphangiogenesis and the development of a functional lymphatic network and/or result in the potential loss of lymphatic identity^35^, thus investigations are needed to assess this. Some of these differences, for example in *FLT4* or *PROX1* expression, could again be due to the immortalization process or due to cell-of-origin variability, making the imLECs here less reliant on *FLT4* or *PROX1* expression for growth compared to HDLECs. Interestingly, when looking at other differentially expressed genes, we found a significant upregulation in *FLT1* and *KDR* (which encodes VEGFR1 and 2) (Supplementary  Tables 1 and  2) and we therefore validated the upregulation of *KDR* through RT-qPCR (Figs. 3D and 4D**).** This upregulation suggests the imLECs may rely differently on the formation of VEGFR3 homodimers or heterodimers with VEGFR2 and activate VEGFC signaling through different downstream pathways^36,37^.

We then utilised gene set enrichment to see what pathways are differentially expressed in imLECs compared to early HDLECs. To do this, we utilised both Reactome, which provides a more mechanistic view of molecular pathways, and KEGG^30–32^, which gives a more disease-orientated perspective. In the early passage imLECs, Reactome shows that the most enriched pathways are associated with extracellular matrix (ECM) processes such as ECM organisation and degradation, as well as several other pathways related to cell signalling. On the other hand, KEGG shows the most enriched pathway is cancer-related (Fig. 3C) which indirectly would imply ECM organization and GPCR signalling as these pathways are often involved in cancer. As a summary, both analyses highlight ECM-related pathways with a role in cell adhesion, migration and structural integrity. When performing the same analysis in the late passage imLECs (Fig. 4C), ECM organisation and degradation were still highly enriched, however, we observed some differences probably reflecting the cell population changing over time. At late passage, imLECs shifted more towards biosynthesis and degradation of collagen pathways, with a more fibrotic and potentially invasive phenotype. This could be due to endothelial-mesenchymal transition (EndoMT) driving the shift in phenotype, leading the cells to behave more like mesenchymal cells or fibroblasts. Interestingly, we also found enrichment of pathways related to neurons, such as axon guidance, something unexpected based on the endothelial origin of the imLECs. However, research suggests that endothelial cells and neuronal cells may use overlapping molecular mechanisms, due to both showing sprouting abilities^38^.

We also performed gene set enrichment analysis specifically on the differentially expressed PLA-associated genes from Figs. 3 and 4, both in the early passage (Supplementary Fig. 2D ) and late passage (Supplementary Fig. 2E ). Both analyses revealed pathways relating to VEGF signalling, which is to be expected due to the observed downregulation of *FLT4* and upregulation of *KDR*. Interestingly, in the samples from late passages of imLECs, we observe several pathways related to gap junction dynamics such as assembling, internalisation and degradation. This could reflect a consequence of the hyperproliferative ability of these cells or could suggest cell lineage drift when taking into consideration the observed ECM-related pathways in the whole gene set enrichment analysis.

As shown in Supplementary Fig. 2A , the PCA places imLECs in two different clusters. We therefore compared the RNA-sequencing data from early and late passage imLECs to identify differentially expressed genes (Supplementary Table 4), followed by a gene pathway set enrichment analysis. This demonstrated significant enrichment in epithelial-mesenchymal transition (EMT), as well as other different signalling pathways such as TNFα-signalling (Supplementary Fig. 2C ), known to induce mesenchymal transition in lymphatic endothelial cells via activation of Activin signals^39^. Together, this suggests that the imLECs may be starting to transform into a more epithelial phenotype, perhaps due to the promotion of cancer-related genes. However, early and late passages of imLECs showed no significant difference in the expression of key lymphatic markers such as *FLT4* or *PROX1*, confirming their expression is maintained over long-term culture (Supplementary Table 4).

While Spearman’s rank correlation revealed a high correlation between samples, DESeq2 analysis highlighted significant differences in their transcriptomes. This could be due to the different parameters the tests are analysing. Spearman’s rank correlation assesses how well the ranks of the two datasets correlate, as opposed to absolute differences. DESeq2 also focuses on individual gene-level changes by performing a statistical test on each gene to identify significant differences between the groups^40^. Thus, it could be that the expression rank is similar between the groups, but there are many genes with large differences in the fold change itself. Either way, how those differences translate into protein expression and functional differences (e.g. VEGFC response) must be investigated.

### VEGFC induces proliferative and migratory responses in imLECs in both 2D and 3D-cellular assays

Functional VEGFR3 expression, a crucial marker for lymphatic cells, and especially its modulation by its cognate ligand VEGFC initiates lymphatic expansion by stimulating proliferation, migration, specialisation and survival. Therefore, when investigating whether lymphatic endothelial cells retain their lymphatic identity and functionality it is essential to assess if expressed VEGFR3 responds to VEGFC^41,42^. RNA-sequencing revealed a 1.38- and 1.23-fold reduction of *FLT4* expression in early passage and late passage imLECs respectively, compared to early passage HDLECs (Figs. 3B and 4B). As previously mentioned, qPCR was used to confirm the downregulation of *FLT4* mRNA expression and an upregulation of *KDR* expression in early and late passage imLECs compared to early passage HDLECs (Figs. 3D and 4D).

To investigate how those differences in RNA expression may relate to protein expression changes, we assessed the expression of VEGFR3 at a protein level and receptor functionality. We used live-cell flow cytometry that verified surface expression of VEGFR3 in approximately 83% of all cell events analysed in late passage imLECs (Supplementary Fig. 1C ), which would confirm functional expression of membrane-localised VEGFR3. Having confirmed surface expression of VEGFR3 in imLECs, we then investigated VEGFC responsiveness of imLECs in several cell-based functional experiments, such as single cell quantitative phase imaging (QPI) to evaluate cellular proliferation and migration, and 3D spheroid-based sprouting abilities.

**Fig. 5:**
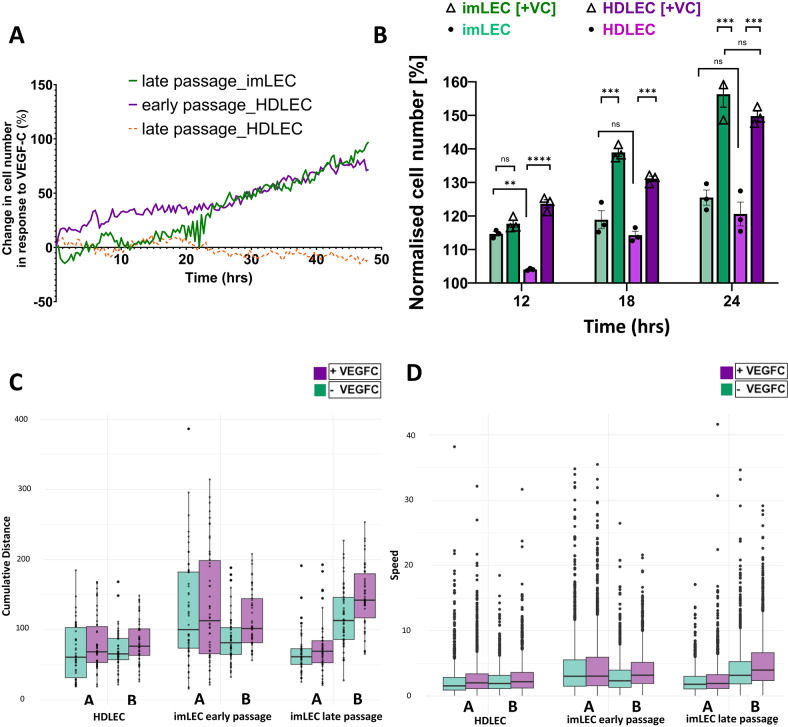
VEGFC driven proliferation and migration in imLECs. (**A**) Plot of single cell count of imLECs (passage 24) versus HDLECs (passage 3 and 7) stimulated with VEGFC (100 ng/mL) over 48 h using Livecyte (Phasefocus). Each data point is captured in 20 min intervals and calculated as Δ number of cells = cell count ^**+VEGFC**^—cell count ^**-VEGFC**^. Normalised data represent mean of *n* = 3 independent experiments. (**B**) Bar chart shows changes in cell number (normalised) in response to VEGFC (VC) (100 ng/mL) stimulation comparing early passage HDLECs (p3) and late passage imLECs (p24) at specific time points of 12, 18 and 24 h. Data represent mean of *n* = 3 independent experiments and error bars indicate mean ± S.E.; ns means nonsignificant and ***p < 0.001; **p < 0.01 and *p < 0.05 significant based on one-way ANOVA with Tukey’s post hoc test or two-tailed unpaired t test. At 12 h time point: imLEC basal vs imLEC +VC (p = 0.127); imLEC basal vs HDLEC basal (p = 0.0022); HDLEC basal vs HDLEC +VC (p = 0.000037). At 18 h time point imLEC basal vs imLEC +VC (p = 0.00044); imLEC basal vs HDLEC basal (p = 0.072); HDLEC basal vs HDLEC +VC (p = 0.00029). At 24 h time point: imLEC basal vs imLEC +VC (p = 0.00013); imLEC basal vs HDLEC basal (p = 0.74); HDLEC basal vs HDLEC +VC (p = 0.000117); imLEC +VC vs HDLEC +VC (p = 0.052). (**C**) Box-and-whisker plot shows cumulative distance cells migrated in HDLECS (passage 3) versus early (p9) and late imLECs (p24) during random migration over 10 h in the presence (purple) and absence (green) of VEGFC (100 ng/mL) in two experimental replicates A and B. Cumulative distance migrated per cell is represented on each plot as a black dot. Mean cumulative distances for HDLEC, early and late passage imLEC with/without VEGFC: 82.0/69.3 µm, 123.7/107.7 µm, 110.9/92.4 µm respectively. (**D**) Box-and-whisker plot shows migratory step-wise speed in HDLECs (passage 3) versus early (p9) and late imLECs (p24) as random migration over 10 h in the presence (purple) and absence (green) of VEGFC (100 ng/mL) for replicates A and B. Mean speeds for HDLEC, early and late passage imLEC with/without VEGFC: 2.83/2.39 µm/sec, 4.26/3.71 µm/sec, 3.82/3.19 µm/sec respectively. The impact of treatment with VEGFC on distance (C) and speed (D) was assessed using the Scheirer–Ray–Hare test; identifying that VEGFC stimulation significantly increased cumulative distance travelled and the speed of cells (P < 0.05).

Firstly, we showed that proliferation of late passage imLECs, measured as change in cell number, was increased when stimulated with 100 ng/mL of VEGFC over 48 h (Fig. 5A, green line) to comparable levels of early passage HDLECs (Fig. 5A, purple line). As expected, late passage of HDLECs showed no proliferative activity in response to VEGFC over 48 h, probably due to mitotic arrest (Fig. 5A, orange line).

Interestingly, we found that early passage HDLECs were more responsive to VEGFC stimulation in the early hours of the experiment, measured by a significant increase in cell number within 12 h, whereas late passage imLECs showed higher rates of proliferative activity at basal conditions (Fig. 5B). Similarly, Frenkel and colleagues reported enhanced proliferation at basal conditions and concluded that imLECs may not require additional VEGFC for proliferation^16^. However, we performed these studies using a higher dose of VEGFC and utilized the advantage of high-content label free imaging to quantify the growth intervals at single-cell level. imLECs exhibited 20% and 30% increase in cell number at 18 h and 24 h, respectively, when stimulated with 100 ng/mL of VEGFC (Fig. 5B), suggesting pro-lymphangiogenic responsiveness of imLECs that was not significantly different from the responses observed in HDLECs at the same time points.

Secondly, we investigated random migratory ability. For each cell type (early passage HDLECs, early and late passage imLECs) we tracked the migration of fifty cells in the presence and absence of VEGFC (migratory tracks shown as rose plots in Supplementary Fig. 3 ) comparing both speed and cumulative distance travelled. For each cell type we found that VEGFC stimulation significantly increased cumulative distance travelled (Fig. 5C) and the track speed (Fig. 5D), with no observed differences in the response between HDLECs and imLECs, while observing differences in the magnitude of the response between the two conducted replicates.

**Fig. 6:**
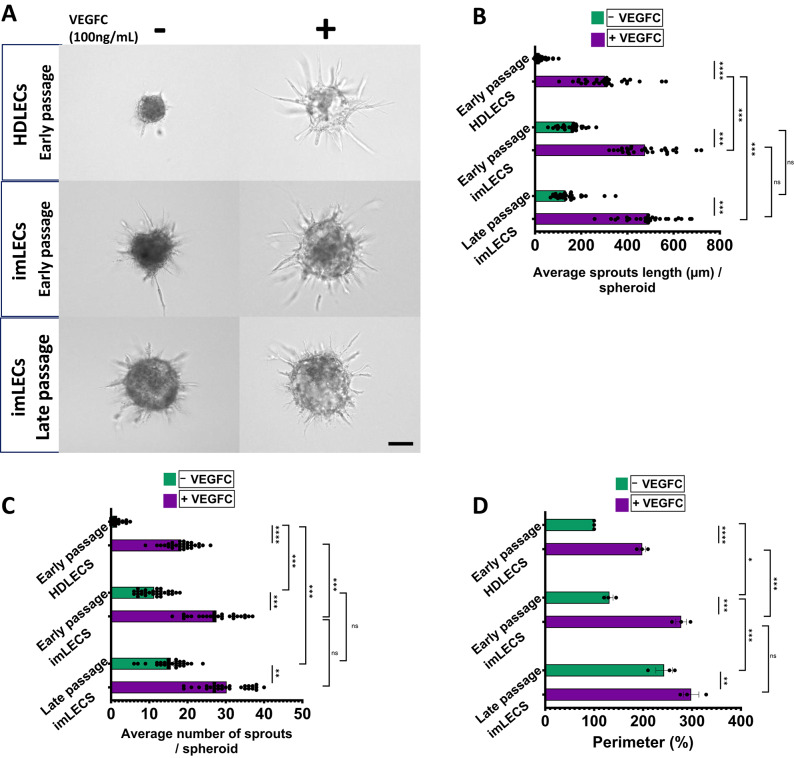
VEGFC-driven sprouting in imLECs. (**A**) Representative images of lymphatic cell sprouts originating from collagen-embedded imLECs (passage 9 and 27) and HDLEC (passage 3) spheroids with or without VEGFC (100 ng/mL). Analysis performed after 24 h using a total of 29 spheroids from 3 independent experiments. Scale bars in all panels, 100 μm. (**B**) Mean average sprouting length (µm) per spheroid. Early HDLEC basal vs early HDLEC +VEGFC (p = 0.000018); early imLEC basal vs early imLEC +VEGFC (p = 0.0005); late imLEC basal vs late imLEC +VEGFC (p = 0.0002); early HDLEC +VEGFC vs early imLEC +VEGFC (p = 0.00076); early HDLEC +VEGFC vs late imLEC +VEGFC (p = 0.00038); early imLEC +VEGFC vs late imLEC +VEGFC (p = 0.17); early imLEC basal vs late imLEC basal (p = 0.077). (**C**) Mean average number of sprouts per spheroid. Early HDLEC basal vs early HDLEC +VEGFC (p = 0.000024); early imLEC basal vs early imLEC +VEGFC (p = 0.00065); late imLEC basal vs late imLEC +VEGFC (p = 0.005); early HDLEC basal vs early imLEC basal (p = 0.00013); early HDLEC basal vs late imLEC basal (p = 0.00044); early HDLEC +VEGFC vs early imLEC +VEGFC (p = 0.00086); early imLEC +VEGFC vs late imLEC +VEGFC (p = 0.073); early imLEC basal vs late imLEC basal (p = 0.059). (**D**) Normalised spheroid perimeter (%) with or without VEGFC (100 ng/mL) comparing early and late passage imLEC spheroids to early HDLEC spheroids. Early HDLEC basal vs early HDLEC +VEGFC (p = 0.000023); early imLEC basal vs early imLEC +VEGFC (p = 0.0008); late imLEC basal vs late imLEC +VEGFC (p = 0.0033); early HDLEC basal vs early imLEC basal (p = 0.042); early imLEC basal vs late imLEC basal (p = 0.00093); early HDLEC +VEGFC vs early imLEC +VEGFC (p = 0.00064); early imLEC +VEGFC vs late imLEC +VEGFC (p = 0.086). Raw (not normalised) data represent mean of *n* = 3 independent experiments and error bars indicate ± S.E.; ns means nonsignificant and ***p < 0.001; **p < 0.01 and *p < 0.05 significant based on one-way ANOVA with Tukey’s post hoc test or two-tailed unpaired t test.

Using a spheroid-sprouting assay, we next investigated the sprouting response to 100 ng/mL of VEGFC over 24 h of early passage HDLECs versus early and late passages of imLECs (Fig. 6A). HDLECs showed enhanced sprouting upon stimulation with VEGFC as demonstrated by the increase of sprout length (Fig. 6B), number of sprouts per spheroid (Fig. 6C) and spheroid perimeter (Fig. 6D) indicating cellular response. All the measured parameters also indicated minimal response in basal conditions (non-VEGFC) in HDLECs. Like HDLECs, both early and late passages of imLECs showed significant increase in all parameters analysed in the presence of VEGFC. However, both early and late passage imLECs exhibit significantly enhanced levels of sprouting length (Fig. 6B), numbers (Fig. 6C) and spheroid perimeter size (Fig. 6D) even in basal conditions compared to HDLECs. In spheroids experiments, responsiveness to VEGFC is also illustrated as fold-change values after data normalised to basal levels in all parameters (Supplementary Fig. 4 ). Increased response in the absence of VEGFC may reflect an intrinsic change due to the immortalisation process or the nature of the cell origin (as reflected in the RNA expression changes we observed by RNA-sequencing) that could make imLECs more responsive to other growth factors present in the cell growth medium, for example Vascular Endothelial Growth Factor 165 (VEGF-165), maybe due to the observed upregulation of VEGFR2^43^.

Overall, we found significant increases in all analysed parameters upon VEGFC stimulation in both early and late passages of imLECs, as we observed in early passage HDLECs. Furthermore, we have observed that the architecture of the 3D sprouts was similar to those exhibited by HDLECs (personal observations). This data, all together, support the usefulness of imLECs as an in vitro model for 2D and 3D proliferation, migration and sprouting experiments as they show a response to VEGFC as seen for HDLECs.

In conclusion, we have shown that imLECs retain long-term expression of lymphatic endothelial markers, including some of the known PLA-associated proteins EPHB4, FOXC2 and ERG, without reaching senescence, at least as seen in HDLECs, marked by an increase in multinucleated cells and an increase in p16 expression (see summary in Table 1). We detected very high correlation in the way imLECs responded to VEGFC, in terms of proliferation, migration and 3D sprouting, compared to early passage HDLECs, indicating that both cell lines are equally suitable models for functional assays.

**Table 1:**
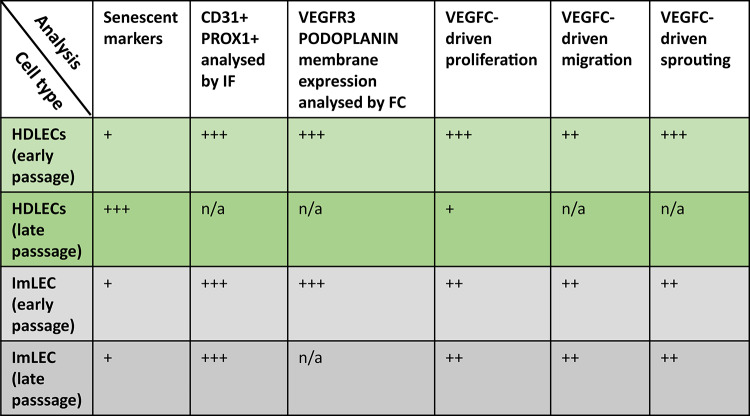
Summary of the results from the functional analyses in imLECs and HDLECs.

CD31 (Platelet endothelial cell adhesion molecule); PROX1 (Prospero homeobox protein 1); VEGFR3 (Vascular endothelial growth factor receptor 3); VEGFC (Vascular endothelial growth factor C); IF (immunofluorescence); FC (Flow Cytometry). + + + indicates > 75% of cells were positive or responded, + + indicates 50–75% of cells were positive or responded, + indicates < 50% of cells were positive or responded, and n/a indicates data not available.

We have also compared early and late passages of imLECs to understand the transcriptional changes resulting from long culture. We expanded the transcriptomic analysis already published by Frenkel and colleagues, by comparing the transcriptomic signatures of early and late imLECs to HDLECs commercially obtained from PromoCell; specifically focusing on genes defined by their inclusion on the PLA diagnostic gene panel and/or their key role in lymphatic development and function. When interpreting the differences observed in our transcriptomic analyses one important consideration is that the imLECs were generated from HMVEC-dLyNeo-Der Lym Endo Cells commercially obtained from Lonza^16^ which could show differences to HDLECs obtained from a different commercial source. It is known that transcriptomic expression in immortalised cells can be different compared to primary cell lines^13,44–46^, in some cases with very low Spearman’s rank correlations^13^. However, this was not the case here, maybe due to the use of BMI-1 as a partner to h-TERT in the immortalisation process^27^.

Throughout the study, some limitations and shortcomings were encountered, including restricted biological replicates in the senescence experiments. This is partly due to time restrictions in collecting such extensive pools of cellular lysates from different starter cell populations. Furthermore, since transcriptional similarities and differences do not necessarily correspond to functional outcome or vice versa*,* to complement transcriptional studies, performing validation studies at the protein level will be beneficiary. In addition to this, VEGFC responsiveness should also be further validated at the protein level by determining VEGFC driven signalling pathways, such as phosphorylated ERKp42/p44 or Akt levels^36,47^.

Different HDLECs batches used in the study were purchased from Promocell isolated from single individuals. Consequently, potential cross donor effects can impact functional assays outcomes and statistical robustness. Moreover, imLECs, created by Frenkel et al.^16^, were originated from human dermal microvascular lymphatic cells, purchased from Lonza. Different companies often use distinct protocols for isolating specific cell populations. With this motivation, we designed this study to investigate and determine the similarities/dissimilarities between HDLECs purchased from Promocell and imLECs in order to expand our understanding on imLEC characteristics and validate their use for future studies.

Moving forward to the use of imLECs in PLA disease modelling, we believe that our findings confirm the suitability of this cell line to assess specific PLA-associated variants, using for example CRISPR/Cas9 gene editing methodologies, due to the replicative capability and proliferation, migration and sprouting responses to VEGFC maintained long-term in culture. However, further investigation is needed on the transcriptomic differences observed especially related to the decrease in *FLT4* expression levels that could impact on downstream signalling analysis.

It is worthwhile mentioning that protocols for lymphatic endothelial cells derived from human induced pluripotent stem cells (h-iPSC) are emerging. These cells have been shown to express specific lymphatic markers such as PODOPLANIN, VEGFR3 and PROX1^48^ and could become an important in vitro cell culture model and serve as a new platform for studying LEC development and patient specific disease modelling. However, protocols for LEC-specific iPSCs are still in their infancy and can be expensive to establish^49^. Therefore, for the time being, imLECs will be a cost-effective in vitro model to advance the molecular and cellular understanding of lymphatic disease mechanisms, based on the observations made in this study.

## Methods

### Cell culture

Primary human dermal lymphatic endothelial cells (HDLECs) (C-12216, PromoCell) and BMI-1 and h-TERT immortalised human microvascular endothelial cells (HMVEC-dLyNeo-Der Lym Endo Cells)^16^ (here referred to as imLECs) were cultured in supplemented endothelial cell growth medium-complete EBM MV2 (C-22022, PromoCell) containing 50 ng/mL VEGFC (9199-VC-025, Bio-techne) on plates coated with fibronectin from human plasma (1 ug/mL Sigma, F2006). In functional studies, 100 ng/mL VEGFC was used to stimulate the cells. All experiments with HDLECs were repeated three times (N = 3) with cells isolated from independent juvenile donors (#4397Z007.2, #440Z009.2, #485Z021.1) by Promocell. For imLECs, experiments were also repeated at least three times on different days (independent technical repeats). Different passages were used for the experiments described below and we refer to: early passages of HDLECs (p3) usually indicating a cell population with a greater proliferative capacity and intact marker expression, late passages of HDLECs (p6-p7) usually indicating reduced proliferative capacity and reduced lymphatic marker expression, early passages imLECs (< p20) and late passages imLECs (> p20). Precise passages used for each experiment are specified in the figure legends.

### RNA preparation and sequencing

HDLECs at p3 (early passage) and p6 or p7 (late passage) and imLECs at p11 (early passage) and p22 (late passage) were plated at 1.5 × 10^5^ per well in 6-well plates and grown for 24 h. Total RNA was isolated using RNeasy (Qiagen) from three (N = 3) experiments. RNA quality and quantity was measured on a NanoDrop spectrophotometer (Thermo Fisher Scientific) and sent to Novogene for paired-end RNA sequencing using Illumina NovaSeq platforms.

### RNA sequencing data bioinformatic analysis and statistics

Contaminating adapter sequences and low-quality bases were trimmed from the FASTQ files using fastp^50^. 90% of bases were over Q30. FASTQ files were aligned to the hg38 reference genome using STAR (version 2.7.11b). Read counting of protein coding exons (Homo_sapiens.GRCh38.91.gtf as reference) was performed by summarizeOverlaps function in GenomicAlignments (version 1.8.4) implemented in R version 4.3.2. Differential expression of Ensembl annotated genes was determined using the DESeq2 package (version 1.46.0) in R with padj < 0.05 considered to be significant. PCA’s were also calculated in DESeq2. Volcano plots were created using ggplot2 (version 3.5.1). Spearman’s rank correlation coefficient was calculated using the R base package and then visualised on a heatmap using the pheatmap package (version 1.0.12). These analyses and plots were produced for all genes or for the 45 genes from the PLA gene panel (Primary lymphoedema (Version 3.11) (genomicsengland.co.uk). Gene set enrichment analysis was performed using the EnrichR webserver^33^ and visualised using Appyters^51^.

### Immunofluorescence

Cells were fixed with 4% (w/v) paraformaldehyde (Sigma-Aldrich) for 15 min, then permeabilised for 5 min with 0.5% Triton X-100 (Sigma-Aldrich) before blocking with 3% (w/v) bovine serum albumin (BSA; Sigma-Aldrich) for 1 h. Cells were incubated with primary antibodies diluted in 1% blocking buffer overnight at 4 °C. Secondary antibodies conjugated to Alexa Fluor dyes (Thermo Fisher Scientific) were diluted 1:500 in blocking buffer and incubated with cells for 1 h at room temperature. Primary and secondary antibodies are listed in Supplementary Table 5. Cells were mounted with DAPI containing Vectashield mounting media (H-1700, VectorLabs). Immunofluorescence was repeated three times (N = 3) and 5 random fields of view (containing 6–10 cells) imaged for quantification. Confocal imaging was carried out using Nikon A1R point scanning confocal microscope at 60X magnification. Images were analysed with ImageJ (NIH).

### Western blot

1 × 10^6^ cells (HDLECs and imLECs) were harvested in lysis buffer (20 mM Tris pH 7.5, 150 mM NaCl, 0.5% Triton X-100) supplemented with protease and phosphatase inhibitors (Sigma-Aldrich). After clarification by centrifugation, protein lysates were separated by SDS-PAGE and transferred to Immobilon-FL PVDF membranes (Millipore). Membranes were blocked with TBS blocking solution (Odyssey Blocking Buffer, LI-COR Biosciences) and incubated with the relevant primary antibody, diluted in TBS blocking solution containing 0.1% Tween-20 (Sigma-Aldrich). After incubation with secondary antibodies diluted in TBS blocking solution containing 0.1% Tween 20 and 0.01% SDS (Sigma-Aldrich), membranes were washed and scanned with the Odyssey infrared imaging system (LI-COR Biosciences). Protein expression was normalized against alpha-tubulin and quantified using Image J. See full list of antibodies in Supplementary Table 5.

### Live cell imaging

Live cell imaging and quantitative analysis of cell growth and migration were performed label-free on the Livecyte system (Phasefocus, Sheffield). 3 × 10^3^ cells (HDLECs and imLECs) in 0.2 ml complete EBM-MV2 medium were seeded per well in 96-well plates (Corning 4580) 4 h prior to imaging. Before imaging, medium was replaced with complete EBM-MV2 containing 100ug/mL VEGFC or fresh complete EBM-MV2. Cells (four randomly selected 1mm^2^ field of view per well) were imaged with Olympus PLN 10X (0.25NA) objective for 48 h at 20 min intervals. For cellular proliferation, high-contrast quantitative phase images were captured using the Livecyte Kinetic Cytometer. Cell count metrics were analysed on Livecyte software before transferring to GraphPad Prism v10 for plotting. For cellular migration, single-cell tracking was acquired by Livecyte Analyse software and data was analysed in R v4.4.2 using celltrackR. Tracks were analysed for 50 cells over time frames 0 ≤ t ≤ 30 (20 min/frame) with speeds (µm/sec) for each cell calculated stepwise for each timepoint. Cumulative distance (µm) was calculated for each cell using the trackLength function^52^. The impact of treatment with VEGFC on cell speed and distance was investigated in R using the Scheirer-Ray-Hare test, a non-parametric extension of a two-way ANOVA, including replicate as a second independent factor, and an interaction term between treatment and replicate.

### Flow cytometry

Single-cell suspensions were prepared from full-grown T75cm^2^ flasks following trypsinization with detach kit (PromoCell) containing Trypsin 0.04%/EDTA 0.03% for 5 min at 37ºC and inactivated with trypsin neutralising solution (PromoCell). Cells were blocked with 3% BSA/PBS and staining for flow cytometry analysis was performed at room temperature with the following fluorochrome conjugated primary antibodies prepared in 1% BSA/PBS for 30 min following two PBS (Ca^2+^ Mg^2+^) washing steps: APC anti-human VEGFR3 (FLT-4) Antibody [Clone: 9D9F9], Brilliant Violet 421 anti-human CD31 Antibody [Clone: WM59] and APC/Cyanine7 anti-Human Podoplanin Antibody [Clone: NC-08] purchased from BioLegend (San Diego, CA, USA) (Full list of antibodies in Supplementary Table 5). Flow cytometry data was obtained on a BD FACS Melody (BD Biosciences) and the data were processed using Kaluza v2.3.1 (Beckman Coulter, USA). HDLECs and imLECs populations were analysed individually.

### Spheroid sprouting assay

To generate spheroids, HDLECs and imLECs (~ 750 cells per spheroid) were trypsinized and suspended in complete EBM-MV2 containing 0.25%(w/v) methylcellulose and seeded in ultra-low adhesive round-bottom 96-well plates (Cat 4515, Greiner, Frickenhausen, Germany). For every biological or technical repeat, a minimum of 30 spheroids were aimed to be generated. After 24 h at 37 °C (5% CO_2_), the quality of the spheroids was visually inspected, and spheroids were harvested. Collagen stock solution was prepared prior to use by mixing on ice 8 volumes (stock 3-4 mg/ml) of rat tail collagen type-1 (Santa Cruz, USA) with 1 volume of 10X HBSS (Gibco BRL, Eggenstein, Germany), and 1 volume of 0.2 N NaOH. Spheroids in neutralised collagen were then mixed with or without 100 ng/mL VEGFC and immediately transferred into prewarmed tissue culture plates. After 30 min at 37 °C, to allow gel polymerisation, 0.5 ml complete EBM-MV2 was pipetted on top of each gel to humidify the collagen. After 24 h, images were taken, and sprouting was quantitated digitally (EVOSM5000) by measuring the average sprout length, number per spheroid and spheroid perimeter.

### Statistical analysis

Statistical analyses, unless otherwise stated, were performed using GraphPad Prism v10 software. Statistical tests included unpaired Student’s t-test and paired t-test to compare two groups, and one-way ANOVA with post hoc Tukey’s for multiple comparisons tests. All statistical comparisons were two-sided. Statistical analysis of sequencing datasets (RNA-sequencing) was carried out using appropriate software packages as defined in the Methods and figure legends. We consider a P value of 0.05 significant. Significance levels of *P < 0.05, ** P < 0.01, ***P < 0.001, **** P < 0.0001 are used throughout.

## Supplementary Information

Below is the link to the electronic supplementary material.

Supplementary Material 1

Supplementary Material 2

Supplementary Material 3

Supplementary Material 4

Supplementary Material 5

Supplementary Material 6

Supplementary Material 7

Supplementary Material 8

## Data Availability

The Livecyte microscopy cell tracking data is available on City St. George’s Figshare ([10.24376/rd.sgul.30075613.v1]). RNA sequencing data are available via European Nucleotide Archive (ENA): study accession PRJEB97700. Details of uncropped western blots source data are available in Supplementary Fig. 5.
