# Supplementary material for: Further characterisation of immortalised human lymphatic endothelial cells to explore their transcriptomic profile and VEGFC response
Source: Sci Rep. 2025 Dec 13;15:45765. doi: 10.1038/s41598-025-28510-8 (PMC12756254; doi:10.1038/s41598-025-28510-8)
Supplement: Supplementary file 5 — Supplementary Material 5 [file 41598_2025_28510_MOESM5_ESM.pdf]

Supplementary figure 4

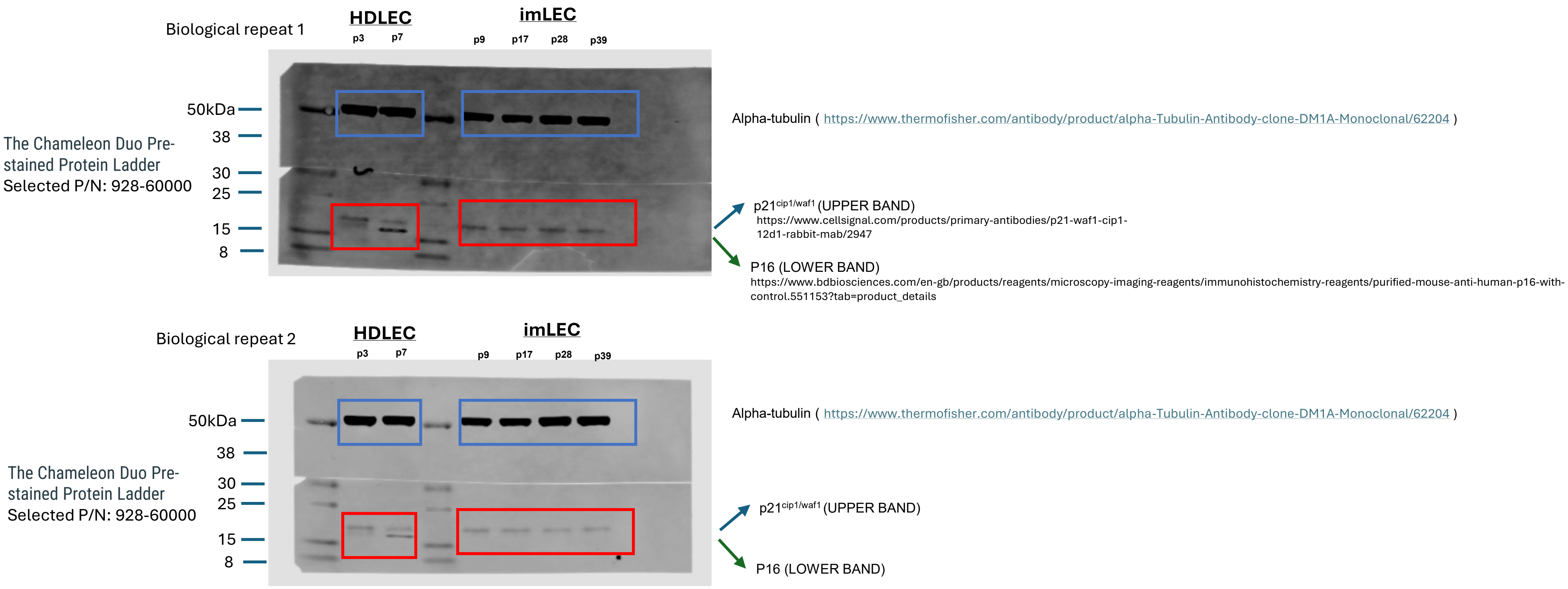

Supplementary figure 4: Uncropped images of 2 independent biological western blots. Web links to previously validated antibodies and protein ladder are provided in the image. Alpha-tubulin bands are indicated in blue boxes at approximately 50kDa range. Both p21 (upper band) and p16 (lower band) are indicated in red boxes.
