# Supplementary material for: Further characterisation of immortalised human lymphatic endothelial cells to explore their transcriptomic profile and VEGFC response
Source: Sci Rep. 2025 Dec 13;15:45765. doi: 10.1038/s41598-025-28510-8 (PMC12756254; doi:10.1038/s41598-025-28510-8)
Supplement: Supplementary file 6 — Supplementary Material 6 [file 41598_2025_28510_MOESM6_ESM.pdf]

SUPPLEMENTARY INFORMATION

Further characterisation of immortalised human lymphatic endothelial cells to explore their transcriptomic profile and VEGFC response

Kazim Ogmen[1], Ruby Moy[1], Sara E Dobbins[1], Lotte van den Bent[2], Onno Kranenburg[2], Jeroen Hagendoorn[2], Alan Pittman[1], Pia Ostergaard[1 ‡], Silvia Martin-Almedina[1 ‡ *]

[1] School of Health & Medical Sciences, City St George's, University of London, London SW17 0RE, UK

[2] Laboratory of Translational Oncology & Dept. of Surgical Oncology, University Medical Center Utrecht, The Netherlands

[‡] These authors jointly supervised this work

[*] Correspondence to Silvia Martin-Almedina, School of Health & Medical Sciences, City St George's, University of London, Cranmer Terrace, London SW17 0RE, UK. E-mail: smartina@citystgeorges.ac.uk. ORCID: 0000-0001-5282-6662.

Supplementary Figure 1

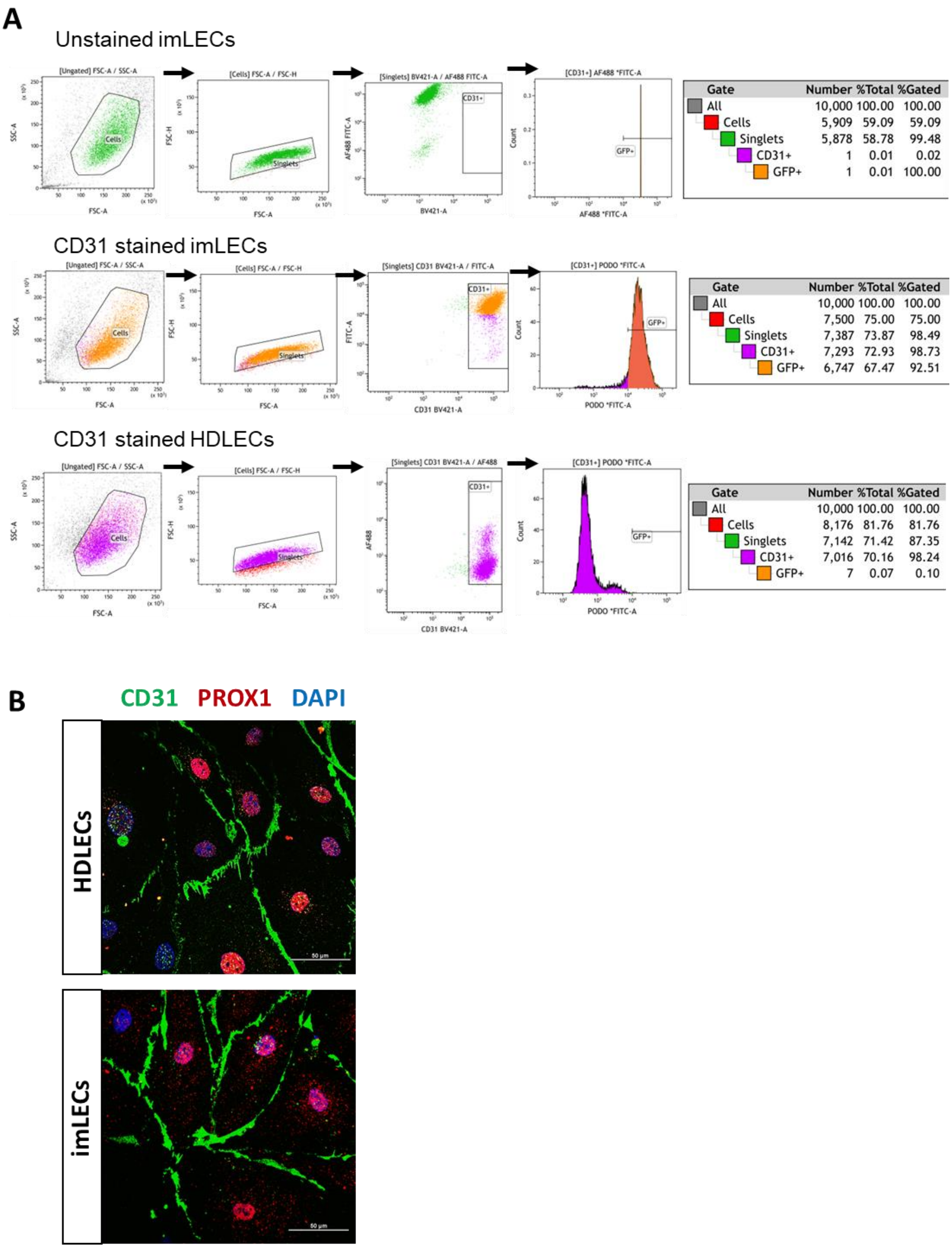

Supplementary Figure 1 (continuation)

C

Unstained imLECs

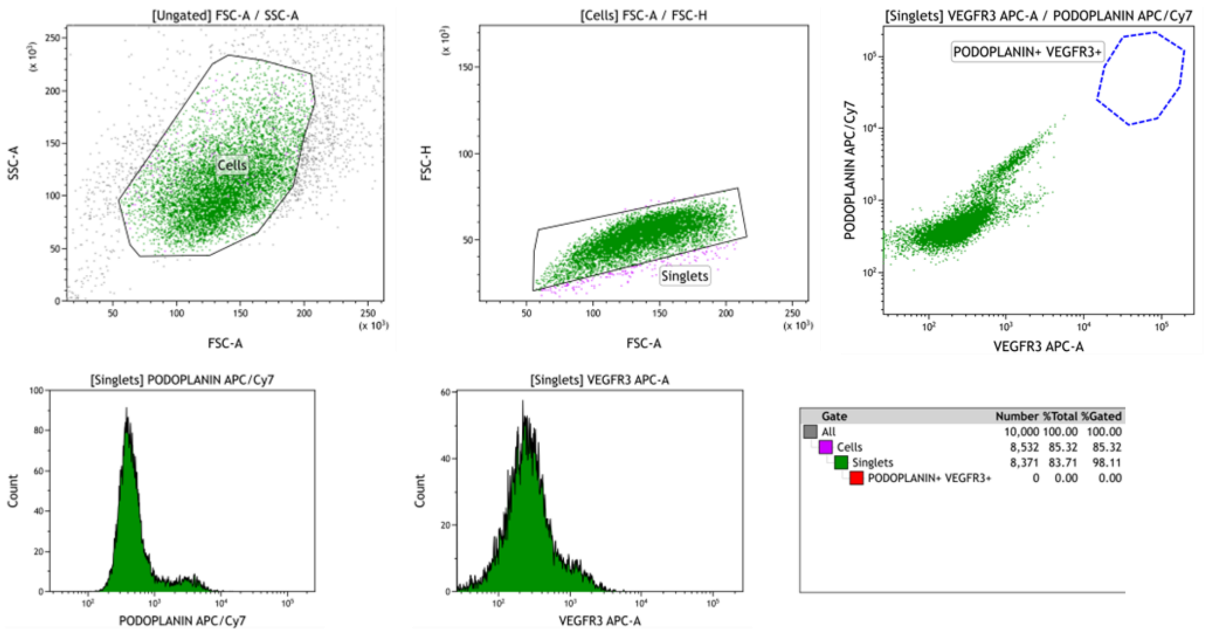

Podoplanin-VEGFR3 stained imLECs

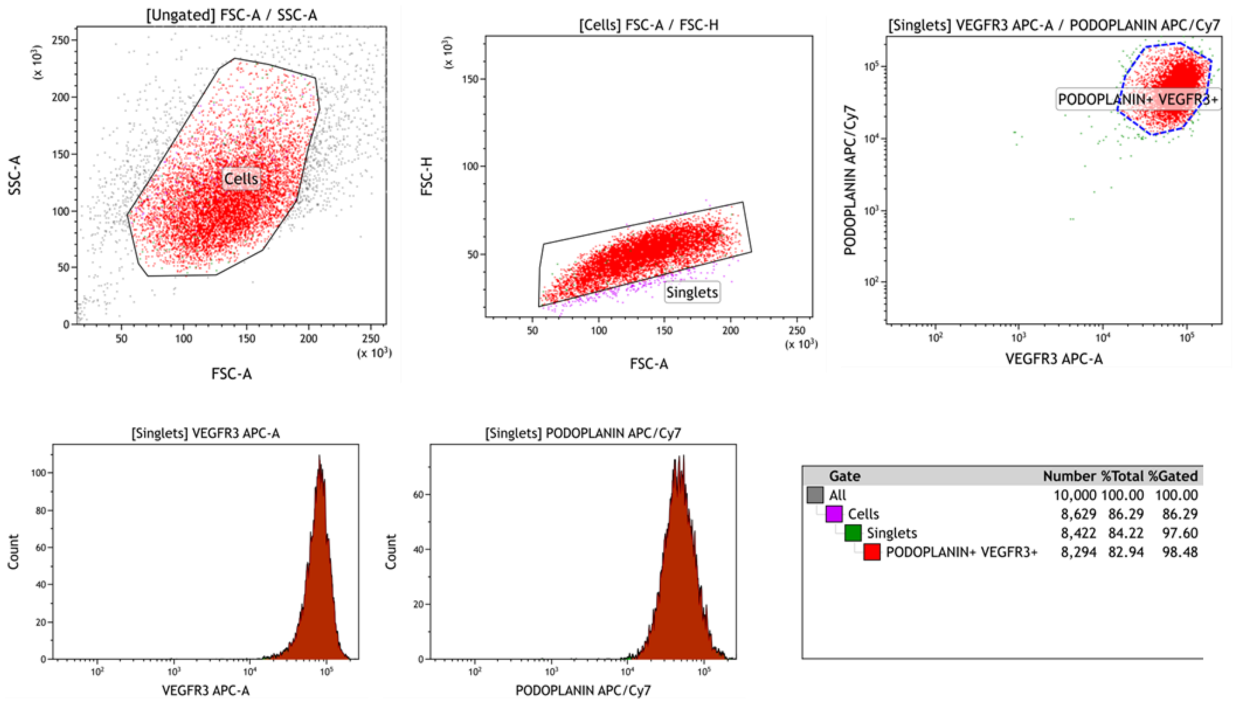

Supplementary Figure 1: GFP+ immortalised lymphatic cells are CD31+, PROX1+, VEGFR3+ and Podoplanin+. A) Sequential gating for flow cytometry analysis of imLECs from passage 9. Initial gating was performed based on forward scatter area (FSC-A) versus forward scatter height (FSC-H) parameters to select single cells for subsequent analysis. imLECs were further analyzed to identify GFP+ and CD31+ cell populations using HDLECs passage 3 as comparison. Data is illustrated as pseudocolour dotplots and histograms. **B)** Subcellular localization of CD31 and PROX1 expression in imLECs (passage 9) and HDLECs (passage 3) was confirmed by confocal microscopy. CD31 receptor was visualized with anti-human CD31 (green) and PROX1 with anti-human PROX1 antibody (red), nuclei with DAPI (blue). Images taken at 60× magnification with a Nikon A1R confocal microscope. Scale bar 50µm. **C)** Sequential gating for flow cytometry analysis of imLECs from passage 9. Initial gating was performed based on forward scatter area (FSC-A) versus forward scatter height (FSC-H) parameters to select single cells for subsequent analysis. Then, imLECs were further analyzed to identify podoplanin+ cells. Within the podoplanin+ cell population, VEGFR3+ cells were identified. Spectrally separated primary antibodies ensured specificity of fluorescent signals. Scatter plot is divided into four quadrants based on differential expression of markers. APC-conjugated anti-human VEGFR-3 (FLT-4) and APC/Cy7-conjugated anti human Podoplanin was used to detect lymphatic phenotype of both cell types. The data is illustrated as pseudocolour dotplots and histograms.

Supplementary Figure 2

A

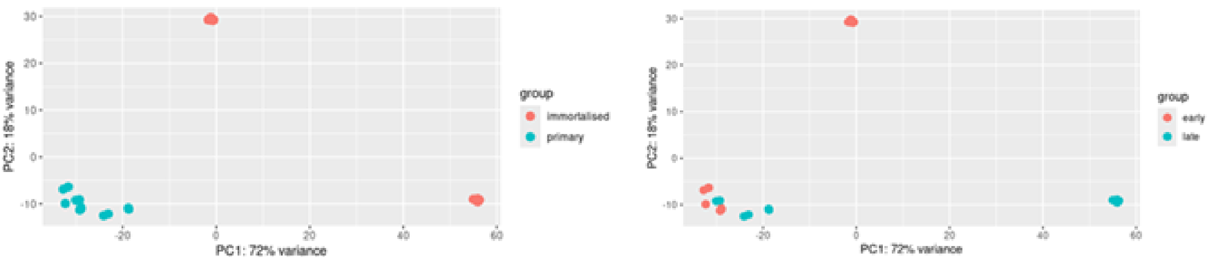

B **Whole transcriptome** **Primary Lymphoedema gene panel**

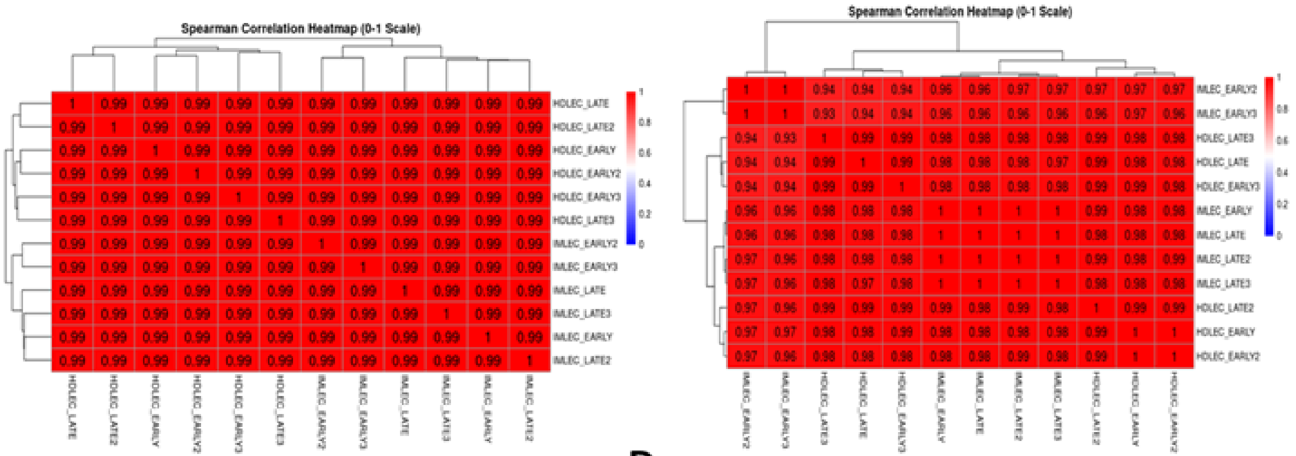

C

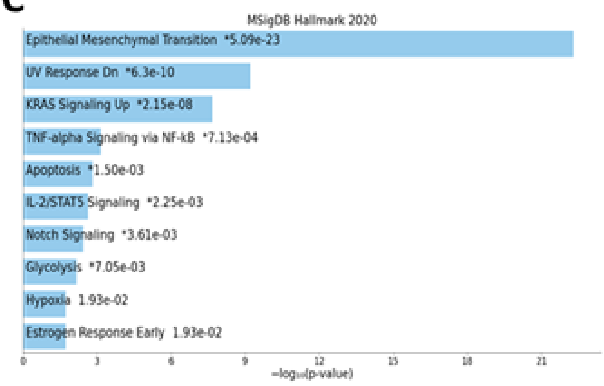

D

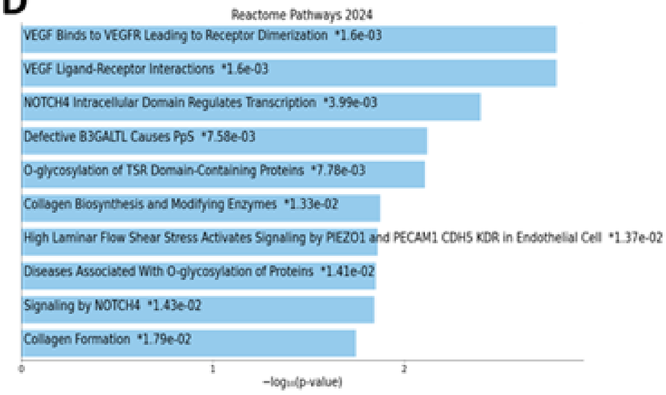

E

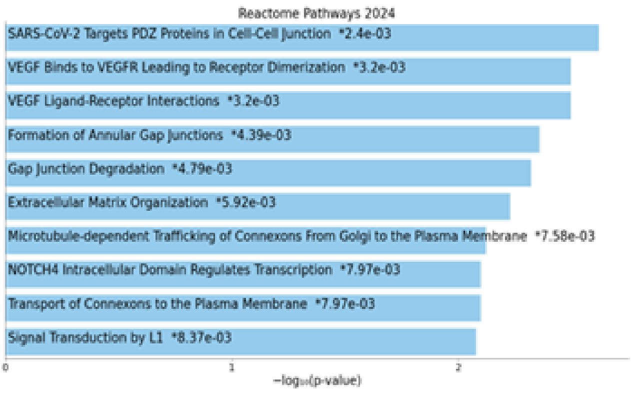

Supplementary Figure 2: Additional Bulk RNA-seq data. A) PCA plots which cluster samples together based on their gene expression profiles. Plots show that there are three main clusters, with all the HDLECs together and the imLECs separating in early passage and late passage. **B)** Heatmap showing Spearman's rank correlation between imLECs both at p11 (early) and p22 (late passages) and HDLECs at p3 (early passage) and p6 (late passage). The Spearman's rank correlation coefficient ranges from +1 indicating a perfect positive, 0 meaning no correlation and -1, a perfect negative correlation. This is represented in the heatmap by colour, with red being a strong positive correlation and blue a relatively weaker correlation. Hierarchical dendrograms show samples that are closely related. Count data was rlog transformed. The heatmap on the left shows the whole transcriptome, with the heatmap on the right showing just genes on the PLA gene panel. The Spearman's rank correlation coefficient for each pair of samples is also written in the square, with 1 being a perfect correlation. **C)** Bar chart of top hits from the MSigDB Hallmark_2020_Human gene set library for imLEC late vs early passages. **D)** Bar chart of top hits from the Reactome human gene set library in imLEC early passages only for the differentially expressed PLA-associated genes from Figure 3. **E)** Bar chart of top hits from the Reactome human gene set library in imLEC late passages only for differentially expressed PLA-associated genes from Figure 4. In all bar charts (C, D and E) the top 10 enriched terms for the input gene set are displayed based on the -log10(p-value), with the actual p-value shown next to each term. The term at the top has the most significant overlap with the input query gene set. All significantly differentially expressed genes were input. An asterisk (*) next to a p-value indicates the term also has a significant adjusted p-value (<0.05).

Supplementary Figure 3

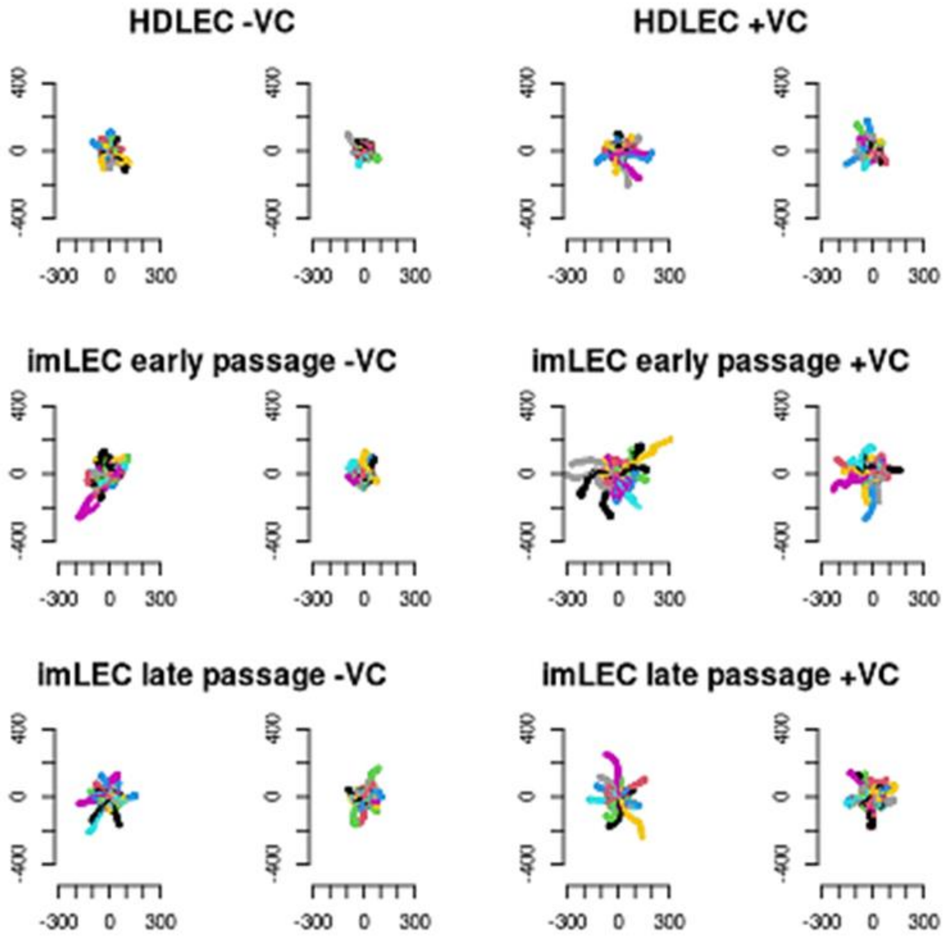

Supplementary Figure 3: Rose plots of imLECs and HDLECs migration tracks. Early passage HDLECs (p3) and early (p15) and late (p29) passage imLECs were subjected to live migration assay with/without 100ng/mL of VEGF in the context of random motility. Single-cell tracking was acquired by Livecyte Analyse software (Phasefocus, Sheffield) and data was analysed in R v4.4.2 using celltrackR. Cell tracks were analysed for 50 cells, with each line representing the track of an individual cell over time frames $0 \leq t \leq 30$ (20min/frame) with displacement (μm) for each cell calculated stepwise for each timepoint and illustrated as rose plots of cell tracks. Track positions were normalized so that each track starts at the (0,0) co-ordinate.

Supplementary Figure 4

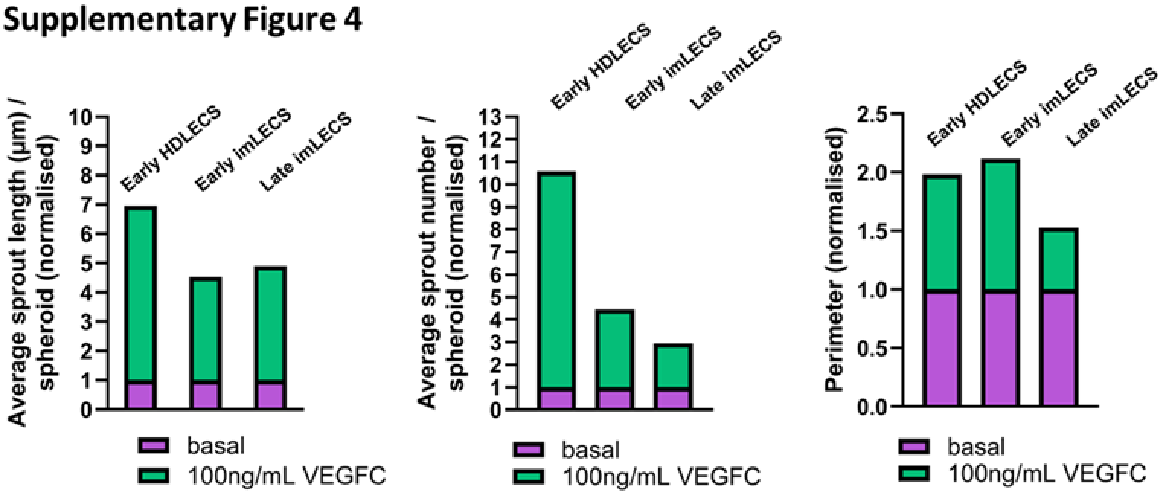

Supplementary Figure 4: Additional quantification of VEGFC-driven spheroid-sprouting in imLECs versus HDLECs. Normalised VEGFC responsiveness in HDLECs and imLECs spheroids were illustrated as fold value changes between all parameters, including average sprout length, sprout number and perimeter of spheroids. Spheroids originating from collagen-embedded imLECs (passage 9 and 27) and HDLEC (passage 3) spheroids with or without VEGFC (100ng/mL). Analysis performed after 24 hours of sprouting using a total of 29 spheroids from 3 independent experiments.

Supplementary Figure 5

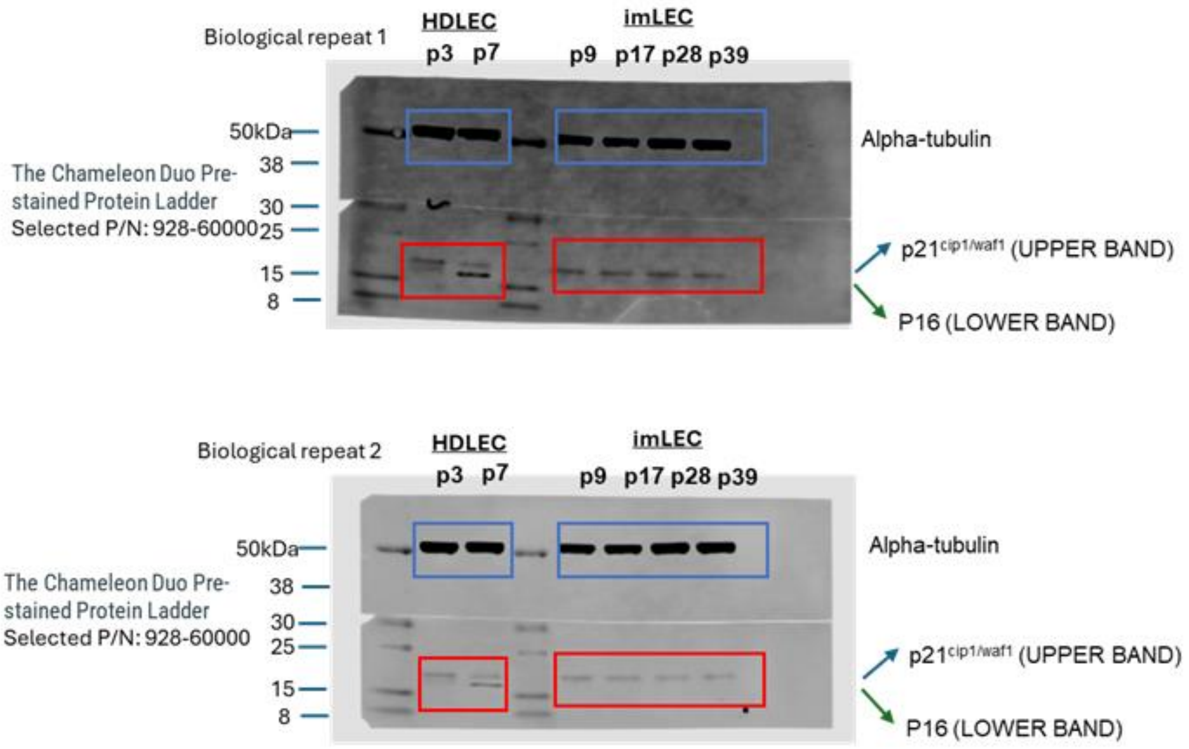

Supplementary figure 5: Uncropped images of western blots from 2 independent biological repeats. Antibodies and protein ladder are provided in the image. Alpha-tubulin bands are indicated in blue boxes at approximately 50kDa range. Both p21 (upper band) and p16 (lower band) are indicated in red boxes. Web links to previously validated antibodies: Alpha tubulin - (https://www.thermofisher.com/antibody/product/alpha-Tubulin-Antibody-clone-DM1A-Monoclonal/62204), p21$^{cip1/waf1}$ –(https://www.cellsignal.com/products/primary-antibodies/p21-waf1-cip1-12d1-rabbit-mab/2947) and p16–(https://www.bdbiosciences.com/en-gb/products/reagents/microscopy-imaging-reagents/immunohistochemistry-reagents/purified-mouse-anti-human-p16-with-control.551153?tab=product_details).

Supplementary Table 1: Table containing DESeq2 differential expression analysis for HDLEC early passage compared to IMLEC early passage.

Supplementary Table 2: Table containing DESeq2 differential expression analysis for HDLEC early passage compared to IMLEC late passage.

Supplementary Table 3: Table containing normalised count data across all samples: HDLEC early, IMLEC early and IMLEC late.

Supplementary Table 4: Table containing DESeq2 differential expression analysis for IMLEC early passage compared to IMLEC late passage.

Supplementary Table 5: Primary and secondary antibodies used in this study.

**Primary Antibodies**	***Source/Company***	***Application***	***Dilution***
anti-Human PROX1 AF2727	Goat / Biotechne	Immunocytochemistry	1:400
anti-CD31 antibody [EPR17260-263]	Rabbit / Abcam	Immunocytochemistry	1:500
anti-VE-cadherin Antibody (C-19): sc-6458	Goat /	Immunocytochemistry	1:500
Anti-ERG antibody [EPR3864]	Rabbit / Abcam	Immunocytochemistry	1:500
anti-FOXC2 (G7) sc-515234	Mouse / Insight Biotechnology	Immunocytochemistry	1:300
anti-Human EPHB4 AF3038	Goat / Biotechne	Immunocytochemistry	1:500
APC/Cyanine7 anti-Human Podoplanin Antibody [Clone: NC-08]	Rat / Biologend	Flow Cytometry	1:100
APC anti-human VEGFR-3 (FLT-4) Antibody [Clone: 9D9F9]	Mouse / Biolegend	Flow Cytometry	1:50
Brilliant Violet 421 anti-human CD31 Antibody [Clone: WM59]	Mouse / Biolegend	Flow Cytometry	1:200
anti-Human p16	Mouse / BD Biosciences	WB	1:1000
anti-p21 Waf1/Cip1 (12D1)	Rabbit / cell signalling	WB	1:1000
Secondary Antibodies	***Source/Company***	***Application***	***Dilution***
Alexa Fluor; Plus 647-1 mg	Mouse / Invitrogen/Life Tech Ltd	Immunocytochemistry	1:1000
Alexa Fluor; Plus 647-1 mg	Goat / Invitrogen/Life Tech Ltd	Immunocytochemistry	1:1000
Alexa Fluor; Plus 488-1 mg	Mouse / Invitrogen/Life Tech Ltd	Immunocytochemistry	1:1000
Alexa Fluor; Plus 488-1 mg	Rabbit / Invitrogen/Life Tech Ltd	Immunocytochemistry	1:1000
Alexa Fluor; 546-1 mg	Mouse /Invitrogen/Life Tech Ltd	Immunocytochemistry	1:1000
Alexa Fluor; 546-1 mg	Rabbit/ Invitrogen/Life Tech Ltd	Immunocytochemistry	1:1000
