# Supplementary material for: Further characterisation of immortalised human lymphatic endothelial cells to explore their transcriptomic profile and VEGFC response
Source: Sci Rep. 2025 Dec 13;15:45765. doi: 10.1038/s41598-025-28510-8 (PMC12756254; doi:10.1038/s41598-025-28510-8)

Supplementary Figures

Supplementary Figure 1

A

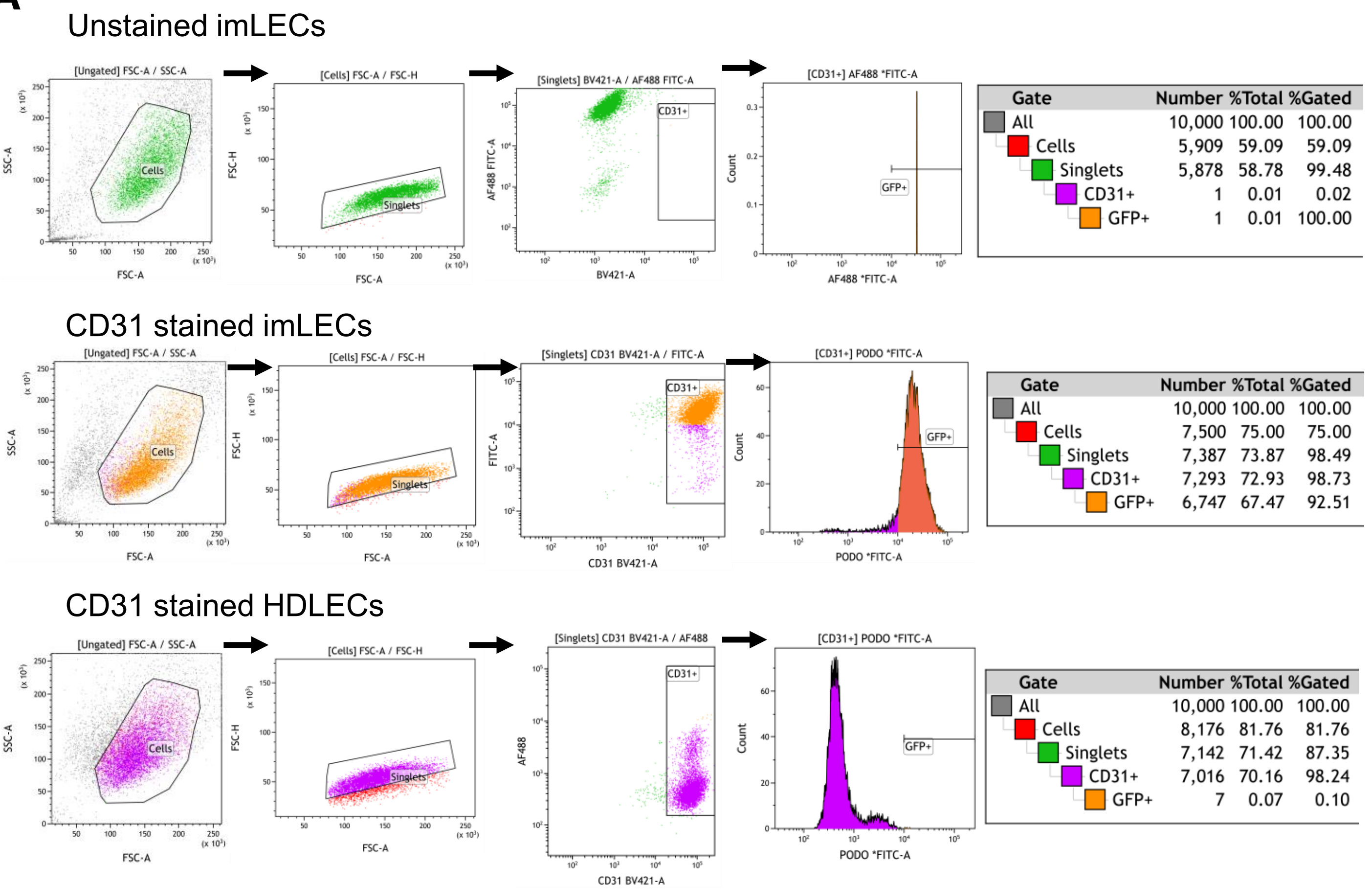

B

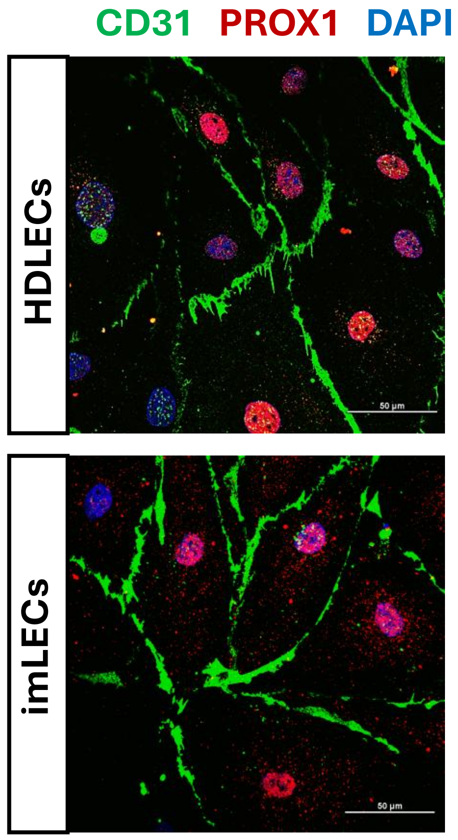

Supplementary Figure 1 (continuation)

C

Unstained imLECs

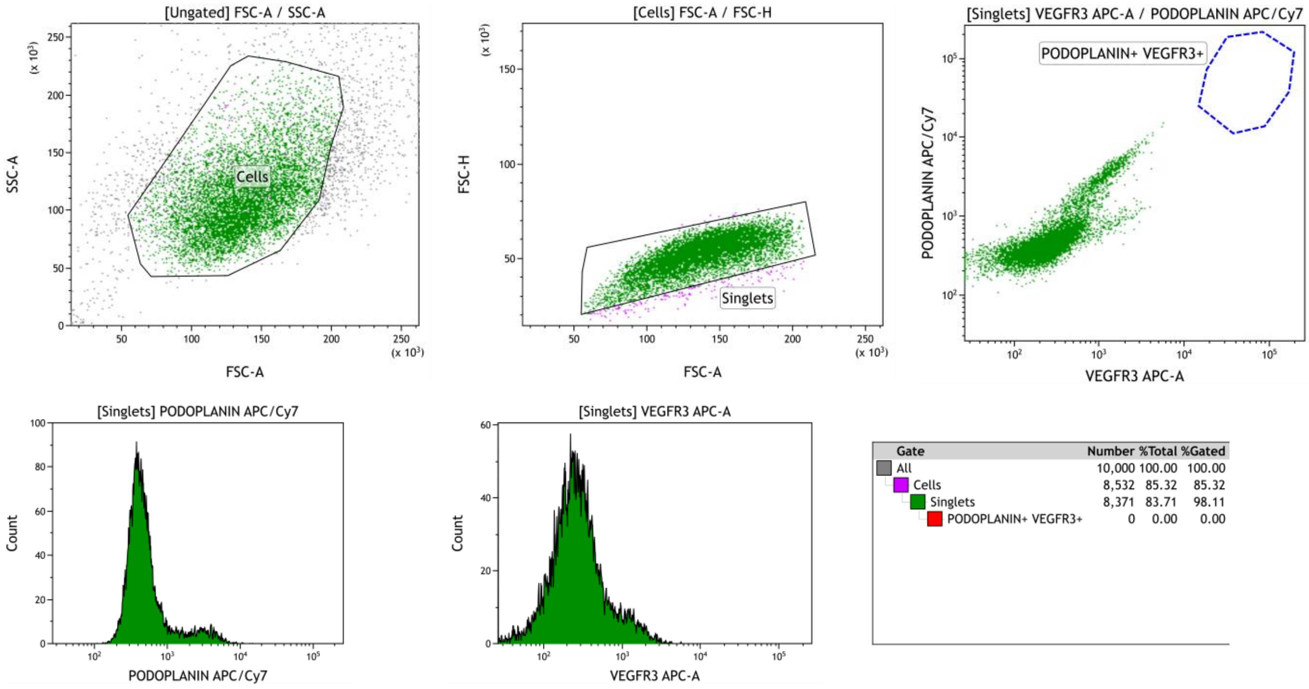

Podoplanin-VEGFR3 stained imLECs

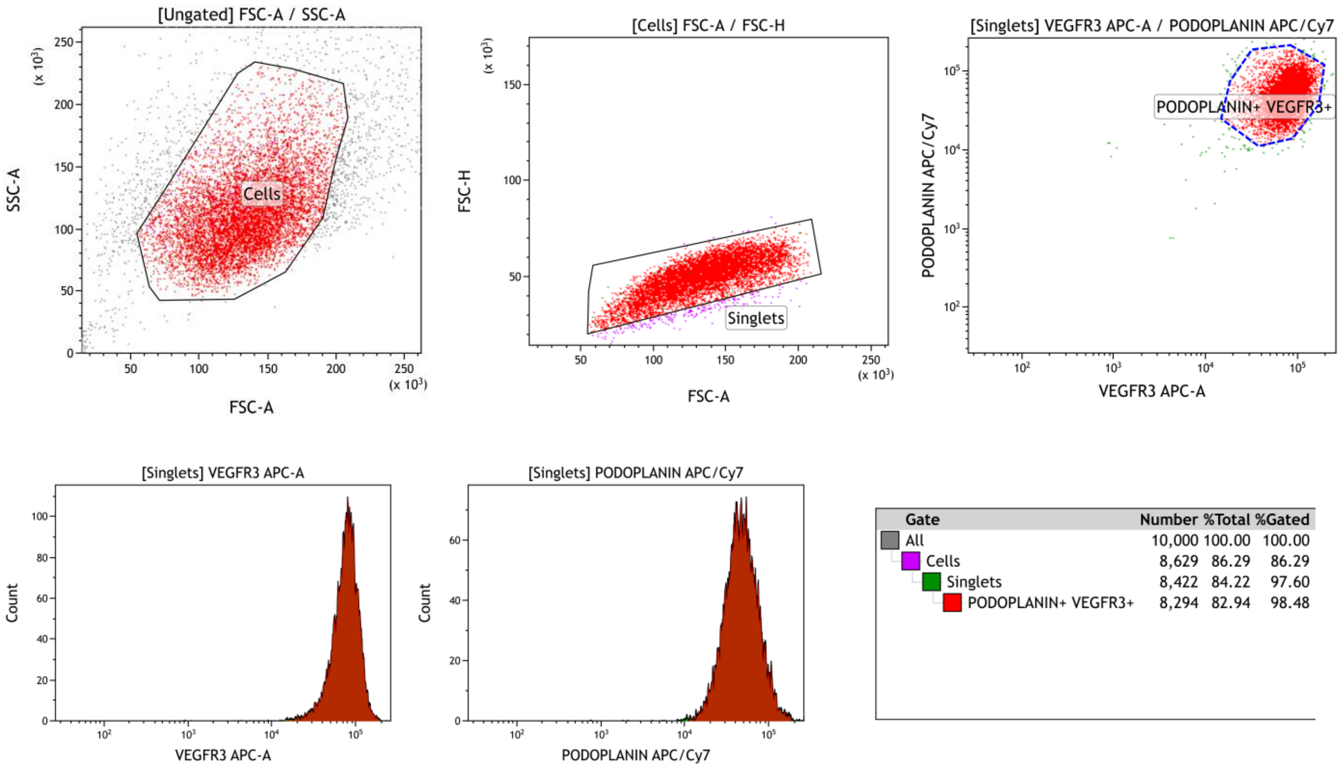

Supplementary Figure 2

A

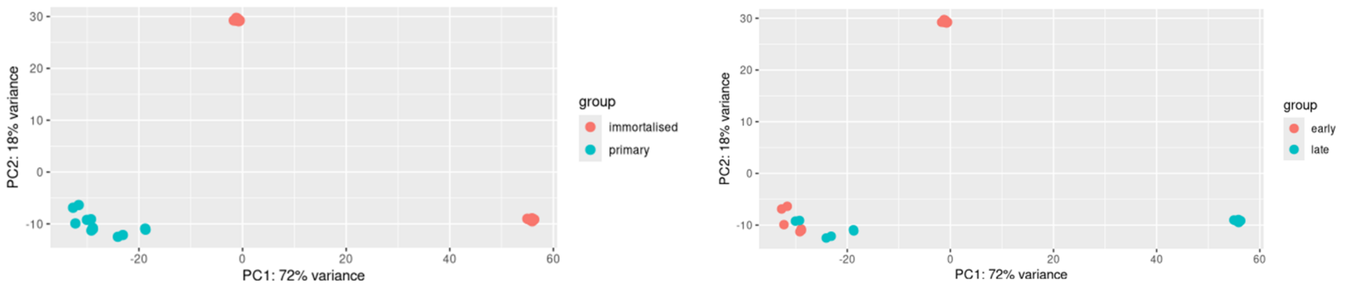

B

Whole transcriptome

Primary Lymphoedema gene panel

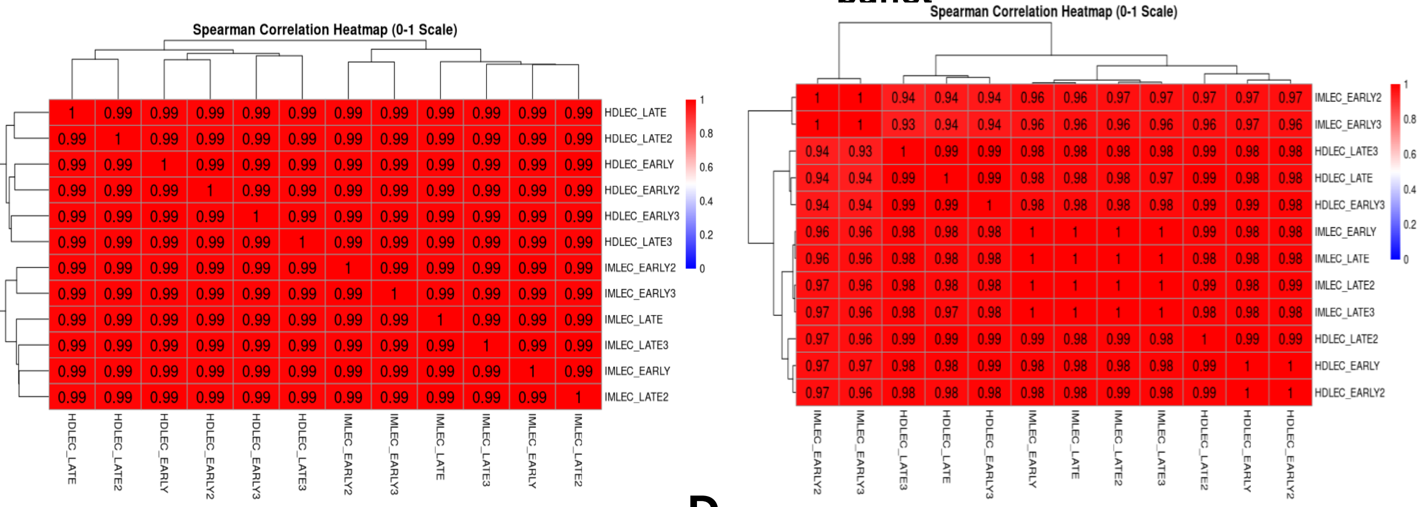

C

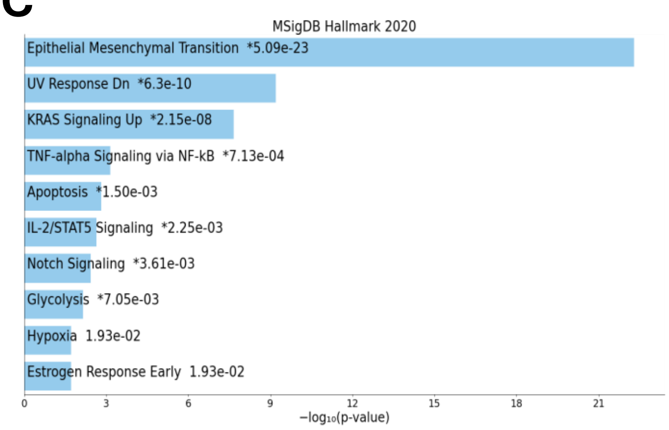

D

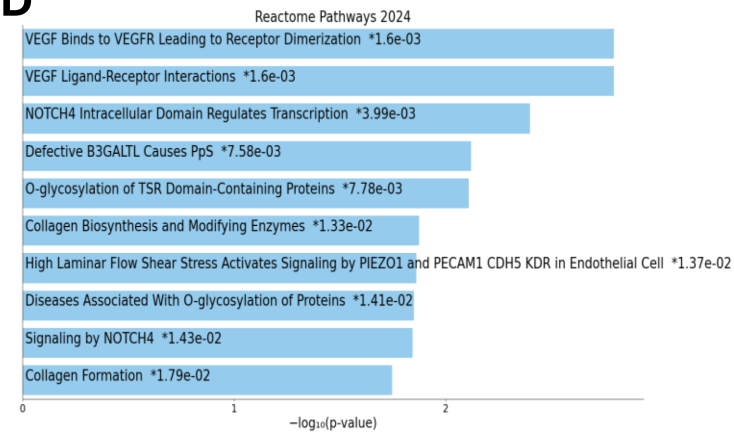

E

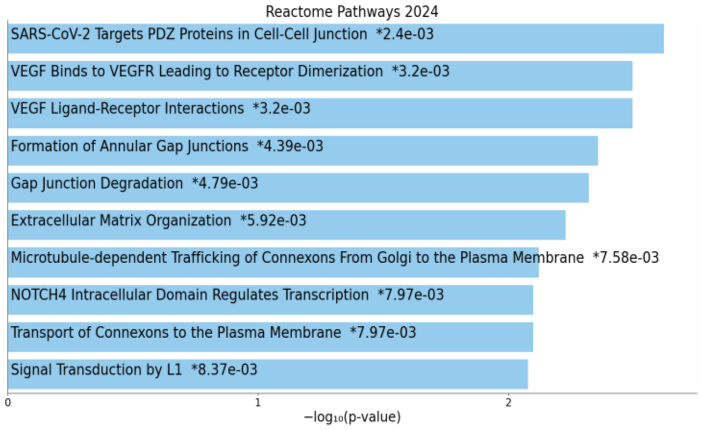

Supplementary Figure 3

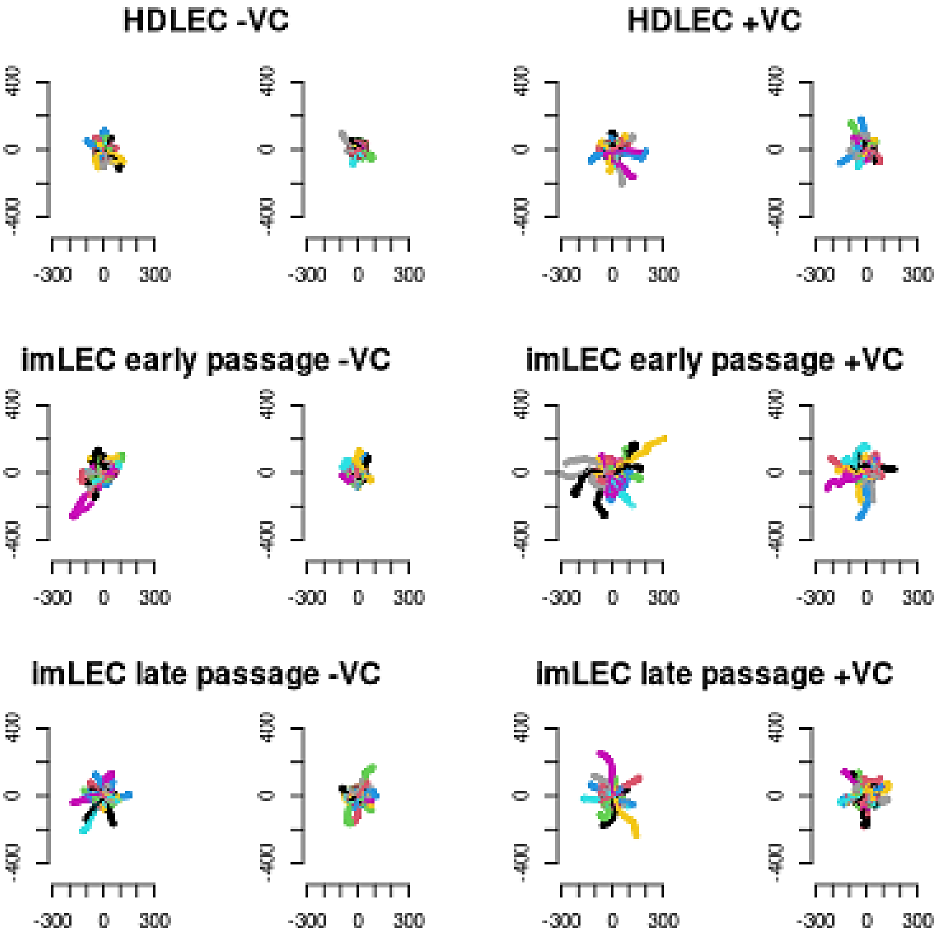

Supplementary Figure 4

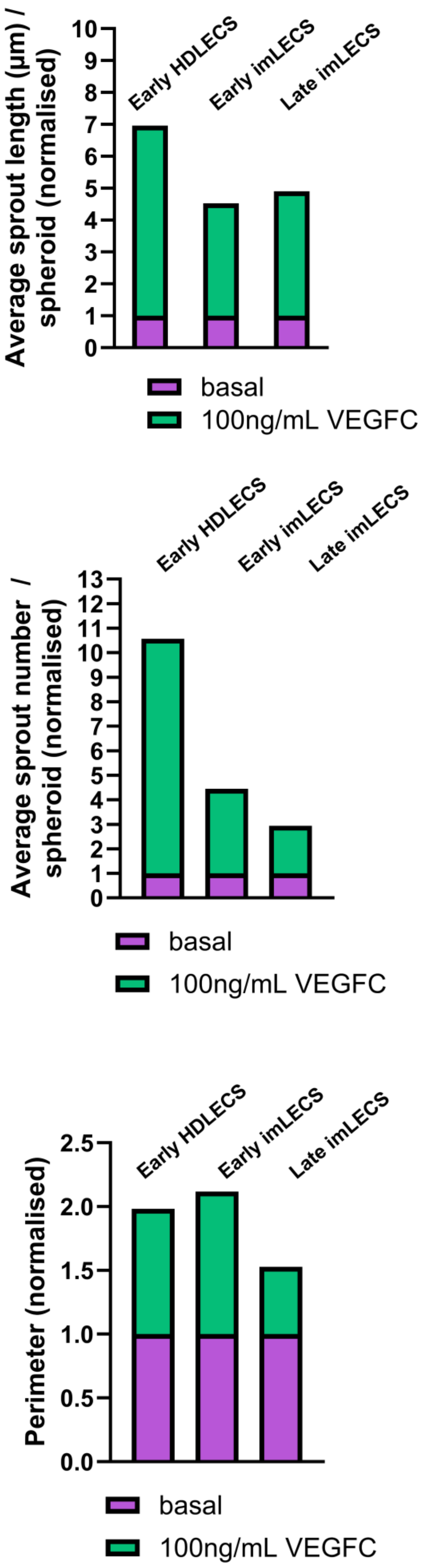

Supplementary Figure 5

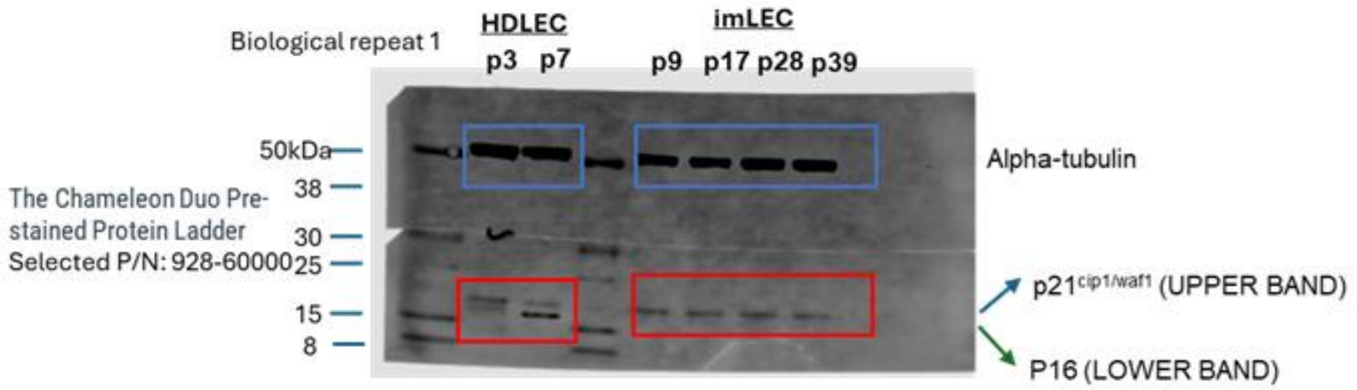

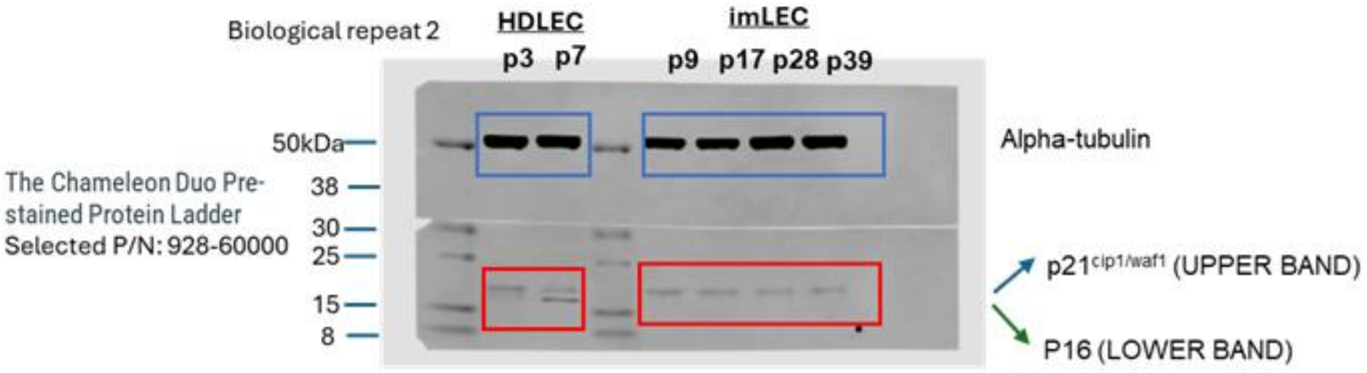

Supplement: Supplementary file 7 — Supplementary Material 7 [file 41598_2025_28510_MOESM7_ESM.pdf]
