# Supplementary material for: Further characterisation of immortalised human lymphatic endothelial cells to explore their transcriptomic profile and VEGFC response
Source: Sci Rep. 2025 Dec 13;15:45765. doi: 10.1038/s41598-025-28510-8 (PMC12756254; doi:10.1038/s41598-025-28510-8)
Supplement: Supplementary file 8 — Supplementary Material 8 [file 41598_2025_28510_MOESM8_ESM.pptx]

## Slide 1
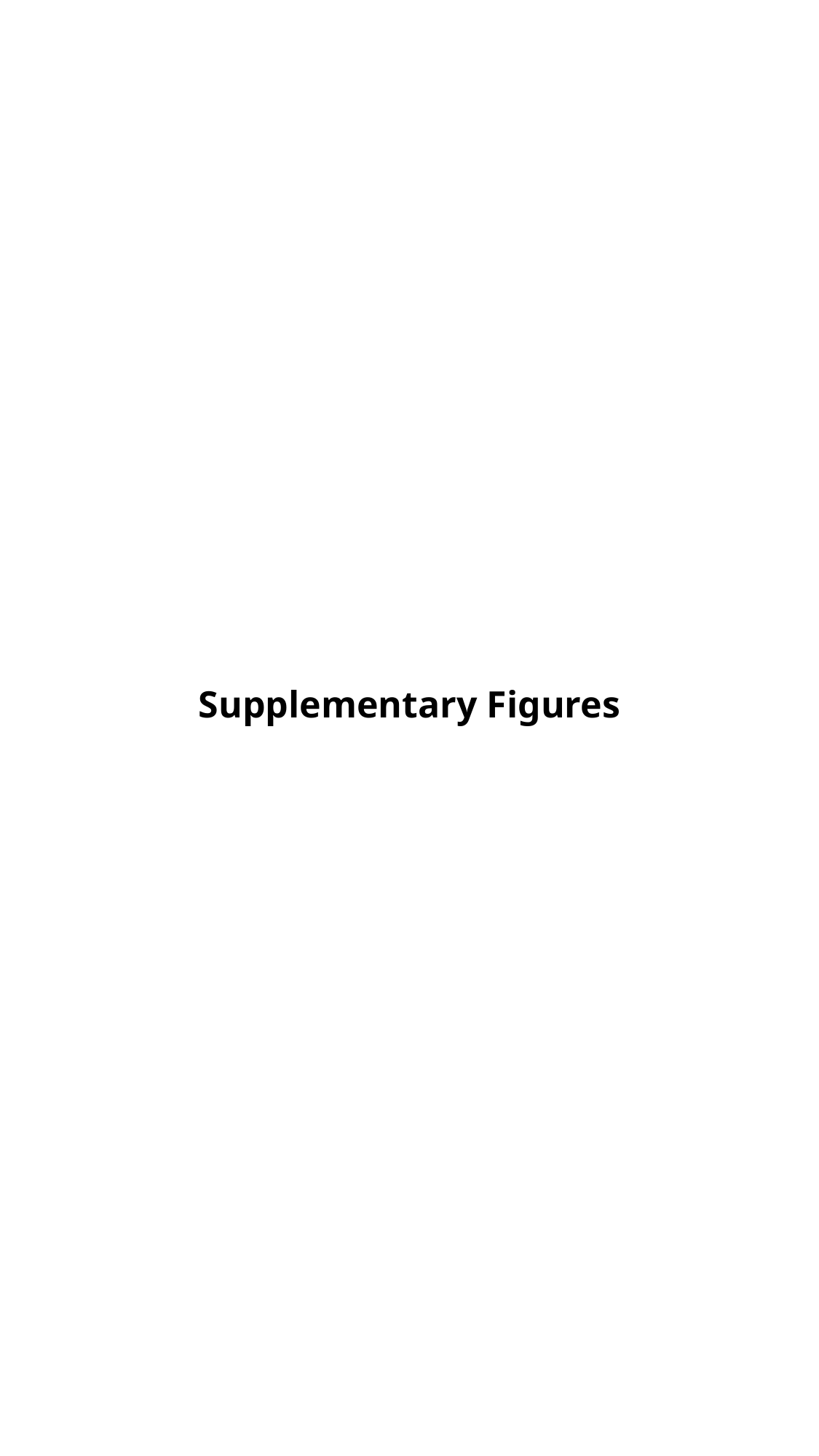

# Supplementary Figures

## Slide 2
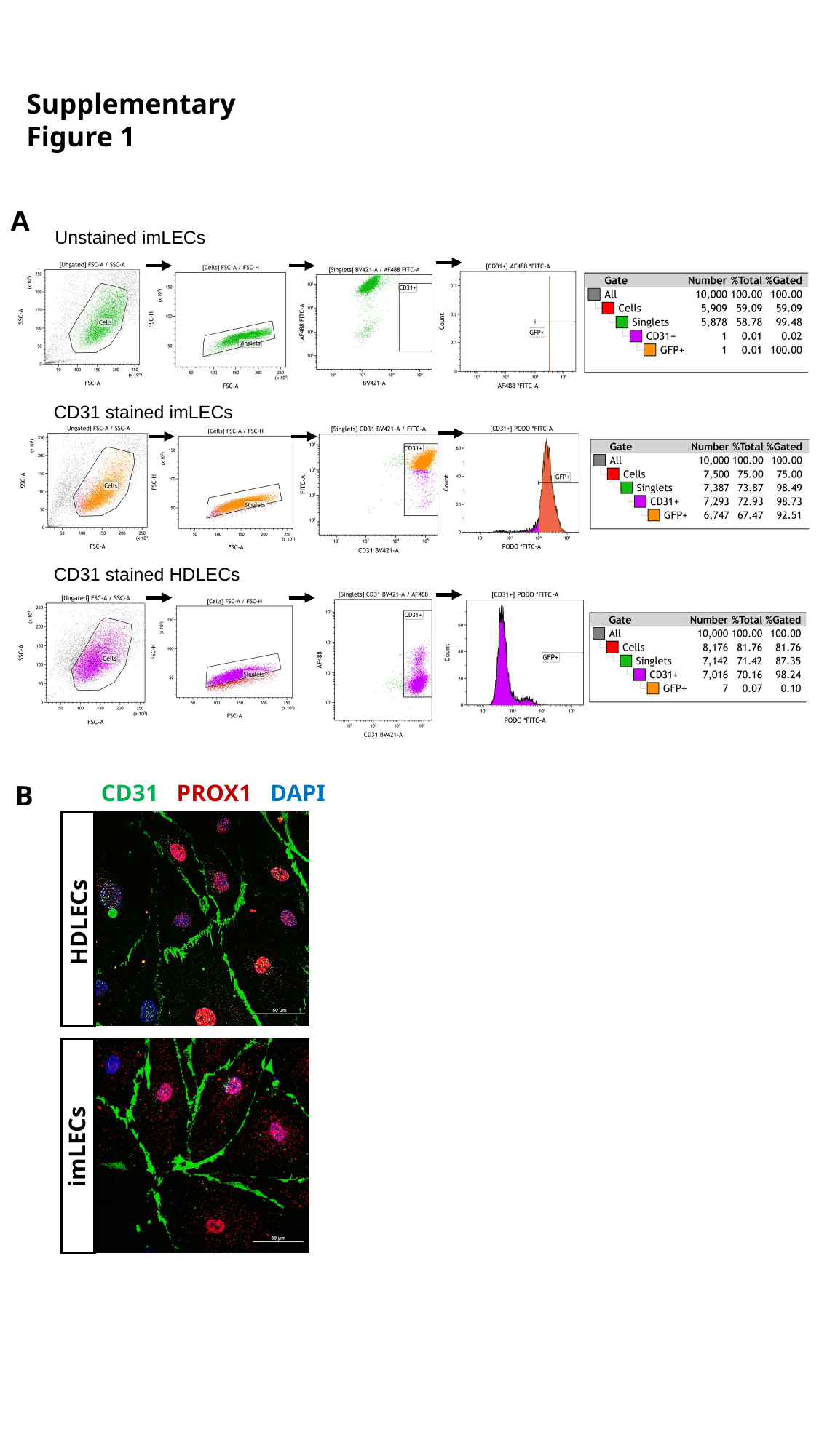

Supplementary Figure 1
A
# Unstained imLECs
CD31 stained imLECs
CD31 stained HDLECs
B
CD31 PROX1 DAPI
HDLECs
imLECs

## Slide 3
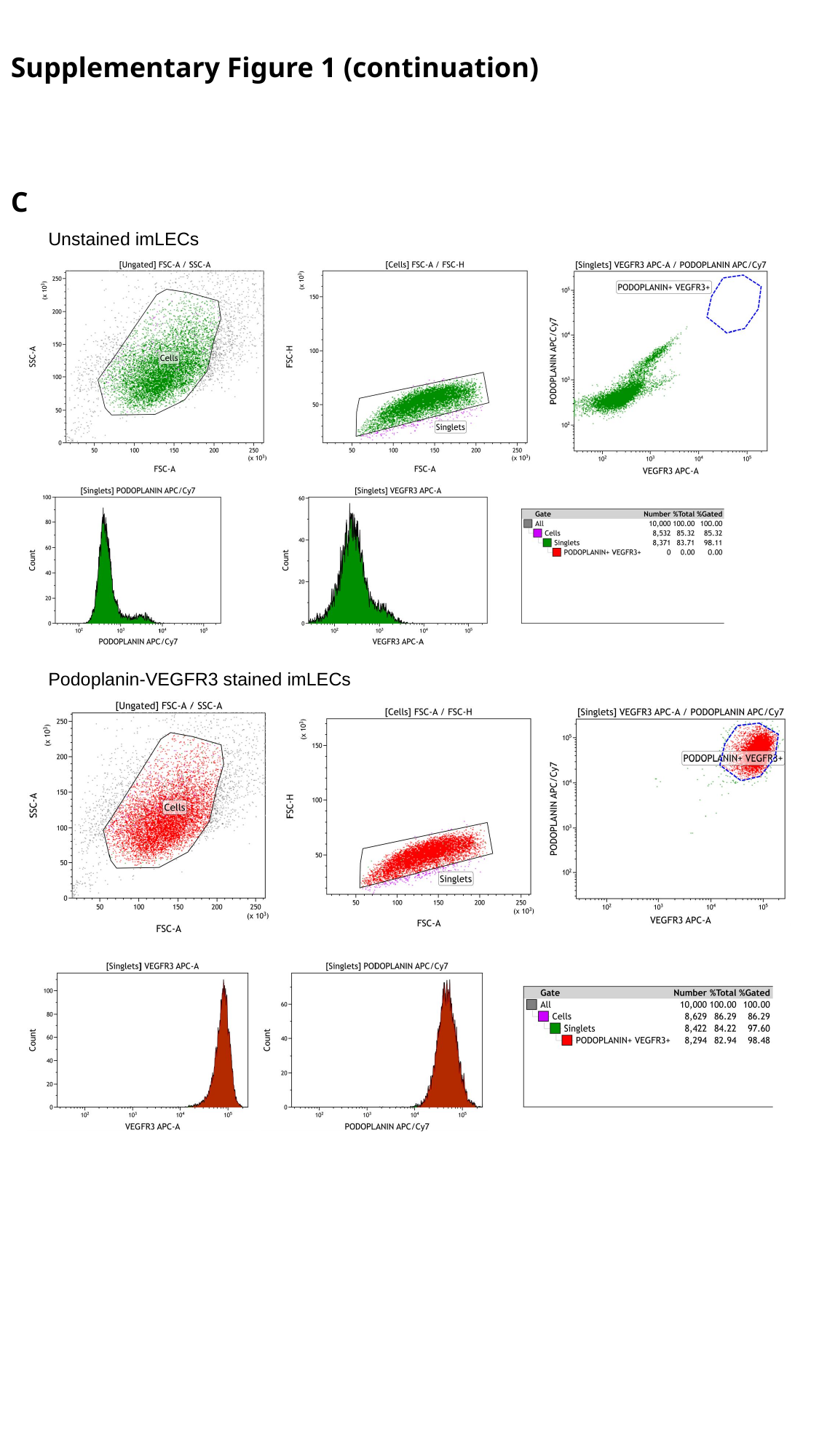

Supplementary Figure 1 (continuation)
C
# Unstained imLECs
Podoplanin-VEGFR3 stained imLECs

## Slide 4
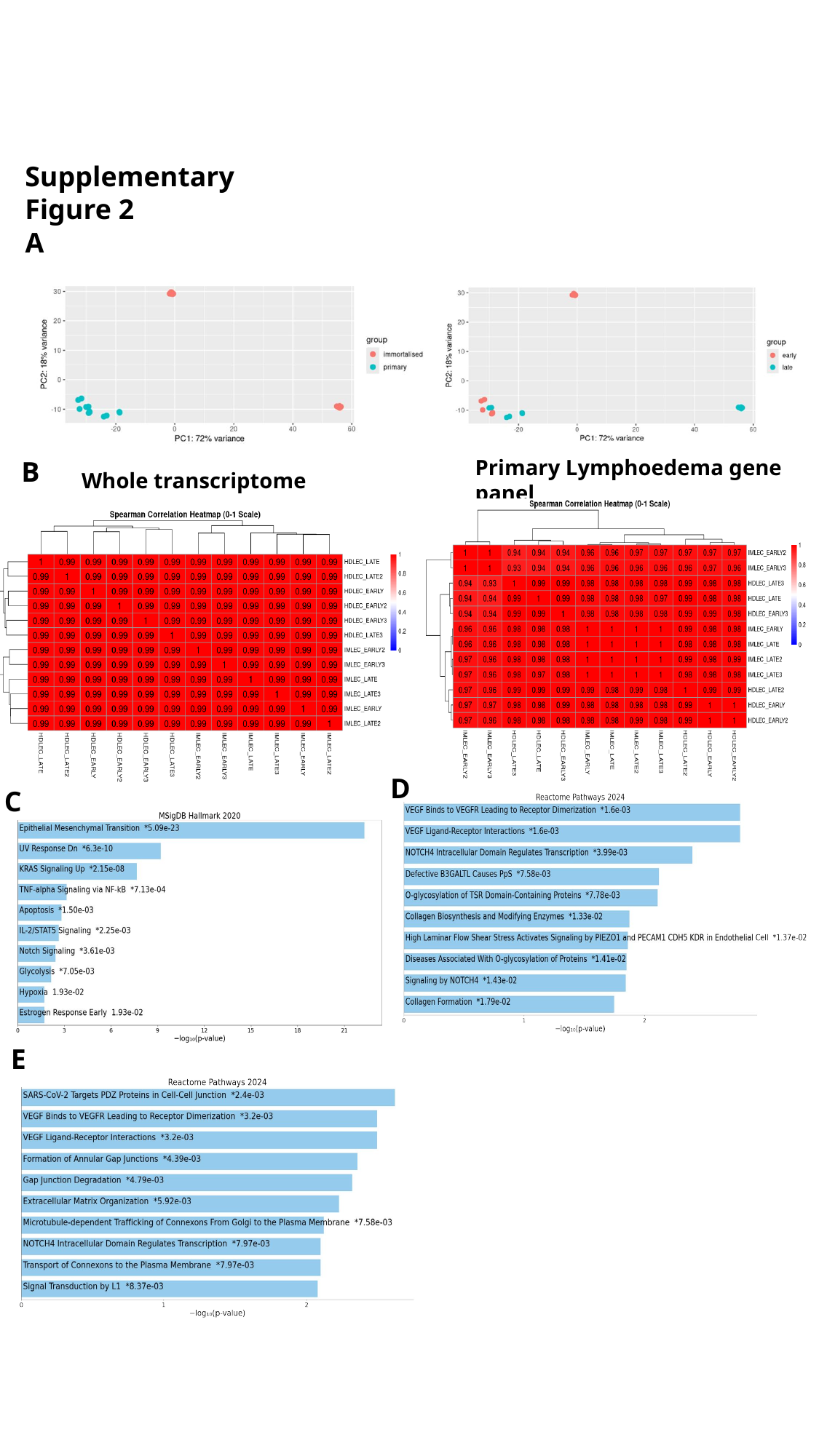

Supplementary Figure 2
A
B
Primary Lymphoedema gene panel
Whole transcriptome
C
D
D
E

## Slide 5
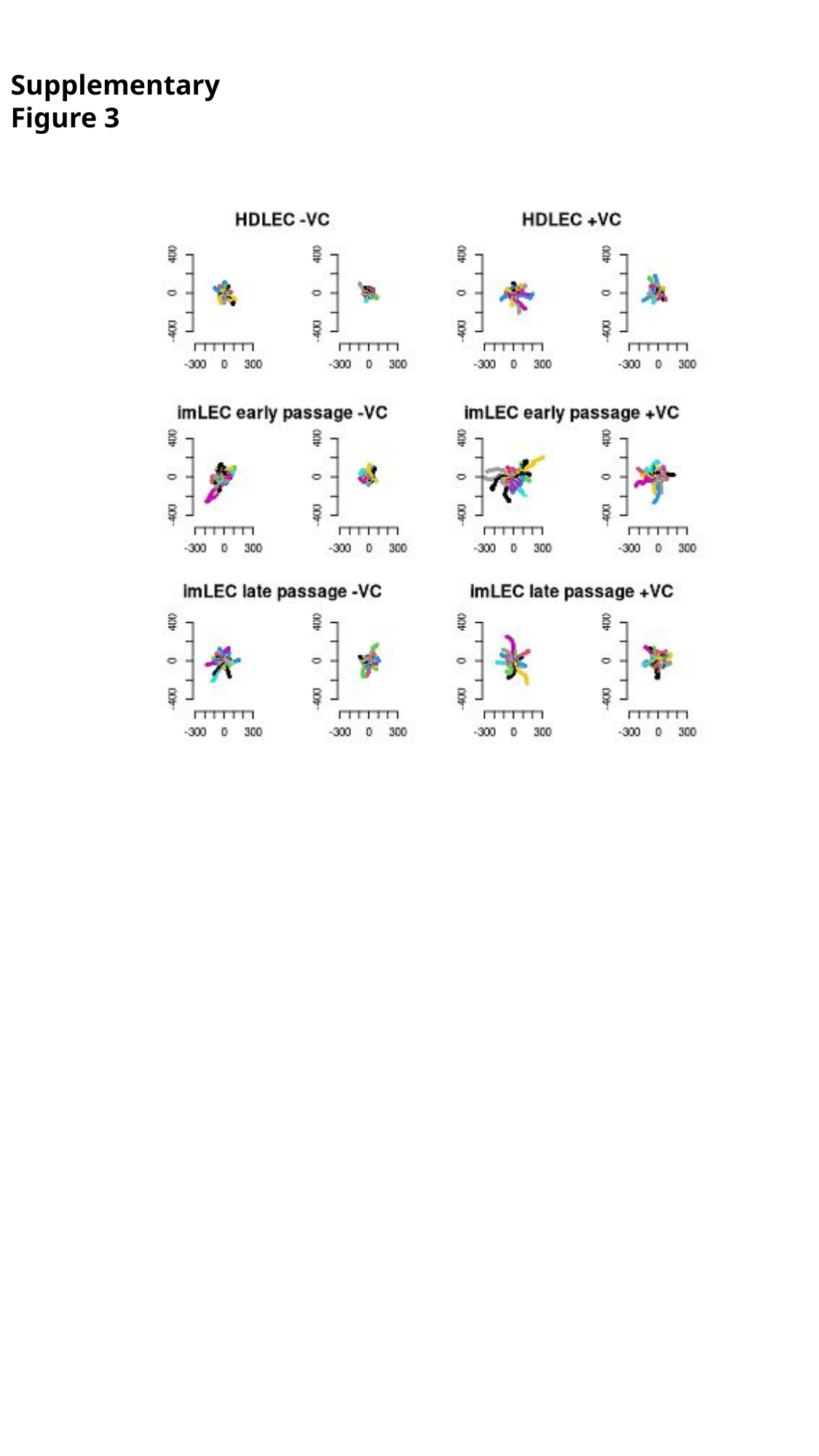

Supplementary Figure 3

## Slide 6
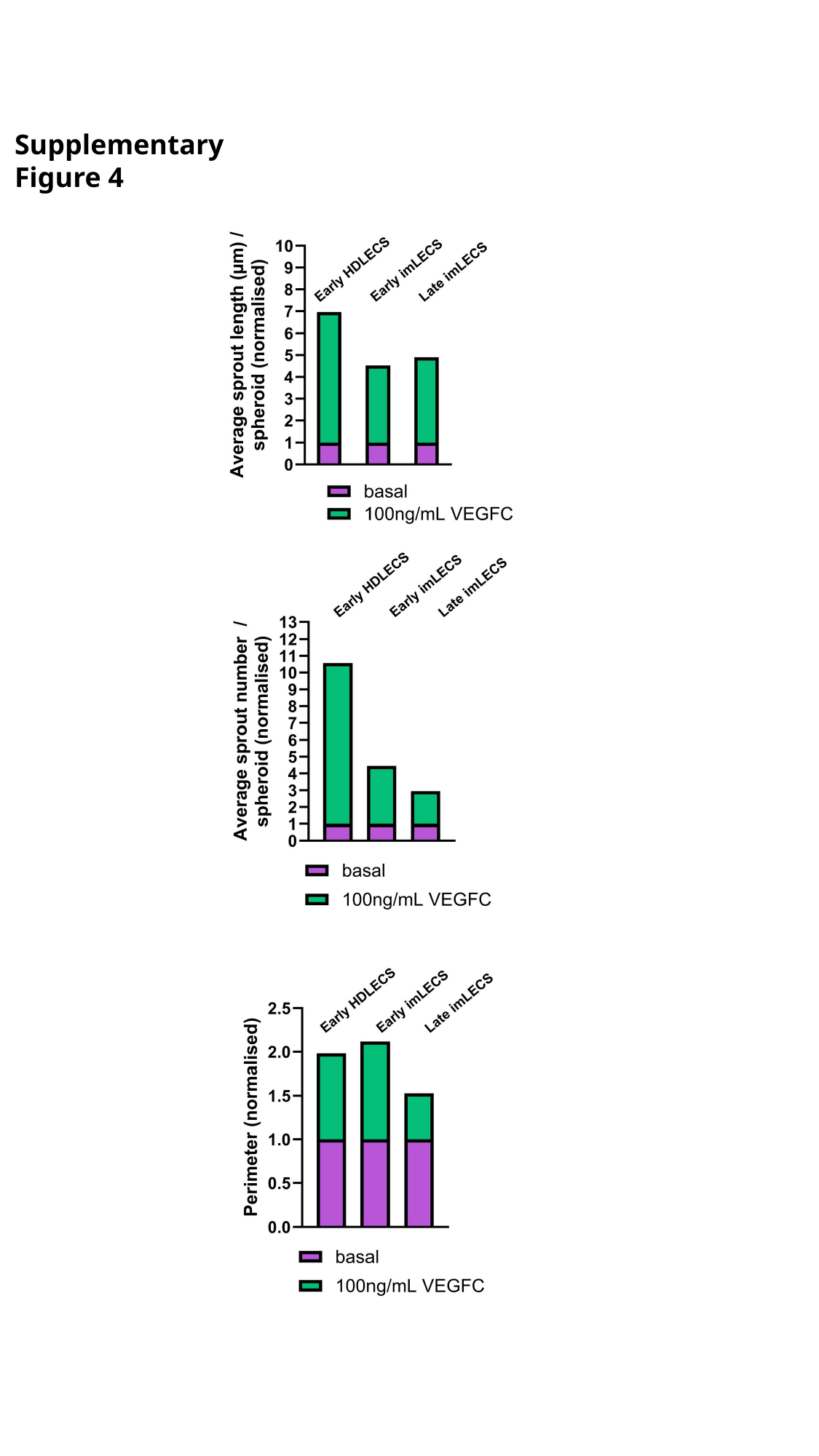

Supplementary Figure 4

## Slide 7
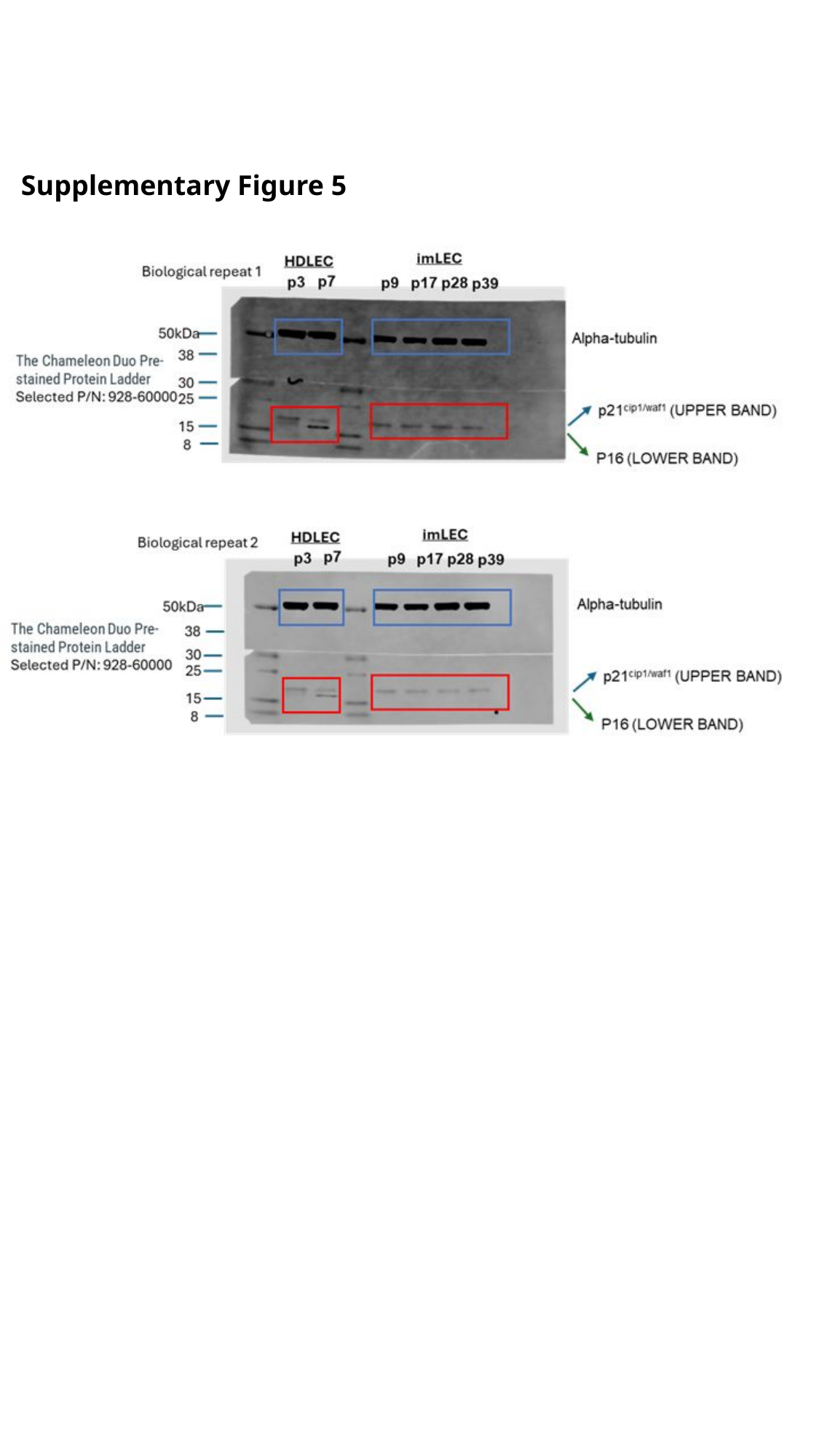

Supplementary Figure 5
